# Systems-level organization of yeast methylotrophic lifestyle

**DOI:** 10.1186/s12915-015-0186-5

**Published:** 2015-09-23

**Authors:** Hannes Rußmayer, Markus Buchetics, Clemens Gruber, Minoska Valli, Karlheinz Grillitsch, Gerda Modarres, Raffaele Guerrasio, Kristaps Klavins, Stefan Neubauer, Hedda Drexler, Matthias Steiger, Christina Troyer, Ali Al Chalabi, Guido Krebiehl, Denise Sonntag, Günther Zellnig, Günther Daum, Alexandra B. Graf, Friedrich Altmann, Gunda Koellensperger, Stephan Hann, Michael Sauer, Diethard Mattanovich, Brigitte Gasser

**Affiliations:** Department of Biotechnology, BOKU - University of Natural Resources and Life Sciences Vienna, Muthgasse 18, 1190 Vienna, Austria; Austrian Centre of Industrial Biotechnology, A-1190 Vienna, Austria; Department of Chemistry, BOKU - University of Natural Resources and Life Sciences Vienna, A-1190 Vienna, Austria; Institute of Biochemistry, Graz University of Technology, A-8010 Graz, Austria; Austrian Centre of Industrial Biotechnology, A-8010 Graz, Austria; School of Bioengineering, University of Applied Sciences FH Campus, A-1190 Vienna, Austria; BIOCRATES Life Sciences AG, A-6020 Innsbruck, Austria; Institute of Plant Sciences, NAWI Graz, University of Graz, A-8010 Graz, Austria; Institute of Analytical Chemistry, University of Vienna, A-1090 Vienna, Austria; Present addresses: Sandoz GmbH, A-6250 Kundl, Austria; Present addresses: BIOCRATES Life Sciences AG, A-6020 Innsbruck, Austria; University of Tübingen, D-72076 Tübingen, Germany

**Keywords:** Metabolome, Methanol, Peroxisome, *Pichia pastoris*, Proteome, Transcriptome, Xylulose-monophosphate cycle

## Abstract

**Background:**

Some yeasts have evolved a methylotrophic lifestyle enabling them to utilize the single carbon compound methanol as a carbon and energy source. Among them, *Pichia pastoris* (syn. *Komagataella sp.*) is frequently used for the production of heterologous proteins and also serves as a model organism for organelle research. Our current knowledge of methylotrophic lifestyle mainly derives from sophisticated biochemical studies which identified many key methanol utilization enzymes such as alcohol oxidase and dihydroxyacetone synthase and their localization to the peroxisomes. C1 assimilation is supposed to involve the pentose phosphate pathway, but details of these reactions are not known to date.

**Results:**

In this work we analyzed the regulation patterns of 5,354 genes, 575 proteins, 141 metabolites, and fluxes through 39 reactions of *P. pastoris* comparing growth on glucose and on a methanol/glycerol mixed medium, respectively. Contrary to previous assumptions, we found that the entire methanol assimilation pathway is localized to peroxisomes rather than employing part of the cytosolic pentose phosphate pathway for xylulose-5-phosphate regeneration. For this purpose, *P. pastoris* (and presumably also other methylotrophic yeasts) have evolved a duplicated methanol inducible enzyme set targeted to peroxisomes. This compartmentalized cyclic C1 assimilation process termed xylose-monophosphate cycle resembles the principle of the Calvin cycle and uses sedoheptulose-1,7-bisphosphate as intermediate. The strong induction of alcohol oxidase, dihydroxyacetone synthase, formaldehyde and formate dehydrogenase, and catalase leads to high demand of their cofactors riboflavin, thiamine, nicotinamide, and heme, respectively, which is reflected in strong up-regulation of the respective synthesis pathways on methanol. Methanol-grown cells have a higher protein but lower free amino acid content, which can be attributed to the high drain towards methanol metabolic enzymes and their cofactors. In context with up-regulation of many amino acid biosynthesis genes or proteins, this visualizes an increased flux towards amino acid and protein synthesis which is reflected also in increased levels of transcripts and/or proteins related to ribosome biogenesis and translation.

**Conclusions:**

Taken together, our work illustrates how concerted interpretation of multiple levels of systems biology data can contribute to elucidation of yet unknown cellular pathways and revolutionize our understanding of cellular biology.

**Electronic supplementary material:**

The online version of this article (doi:10.1186/s12915-015-0186-5) contains supplementary material, which is available to authorized users.

## Background

Methylotrophic yeasts accept a broad range of carbon sources. Multicarbon sources, such as sugars and sugar alcohols like glucose, glycerol, or mannitol, are utilized at similar efficiency as reduced C1-compounds like methanol [1]. Besides the proper equipment of the cells with enzymes necessary for substrate metabolism, their coordinated expression is a prerequisite for the successful utilization of different carbon and energy sources. The methylotrophic yeast *Pichia pastoris* (syn. *Komagataella sp.*) is widely used for recombinant protein production with several biopharmaceuticals on the market [2] and an expanding portfolio of industrial enzymes produced [3]. Recently, the application of *P. pastoris* as a model system for peroxisome and secretory organelle proliferation has also expanded [4, 5]. The methylotrophic lifestyle has been the main driving force for this development, as it involves strong and regulated promoters used for expression of recombinant genes [6], as well as specialized organelles, the peroxisomes. Peroxisomes are defined as intracellular compartments accommodating hydrogen peroxide (H_2_O_2_) forming oxidases together with the H_2_O_2_ detoxifying enzyme catalase. Also the fatty acid beta-oxidation pathway of *P. pastoris* is located in these organelles [7]. Yeast peroxisomal oxidases are predominantly involved in the metabolism of various unusual carbon and nitrogen sources (e.g. alcohols, fatty acids, D-amino acids, or primary amines) [8]. In methylotrophic yeasts, peroxisomes, which harbor the initial steps of the methanol utilization pathway, are highly abundant in methanol-grown cells but become heavily decreased in both number and volume upon catabolite repression [9]. When grown on glucose, *Hansenula polymorpha*, another methylotrophic yeast, harbors only a single, small peroxisome which can serve as a source for proliferation by fission when induction is triggered by shifting the cells to methanol [10, 11]. In addition to genes encoding structural peroxisomal proteins, the expression of methanol utilization related genes is strongly induced on methanol. The first steps of methanol assimilation involve an alcohol oxidase (AOX) to convert methanol to formaldehyde, and a special transketolase named dihydroxyacetone synthase (DAS) to form a C-C bond with the C1 molecule formaldehyde. The reactions of these two enzymes and their localization to peroxisomes are well described [12, 13]. The further reaction cycle of methanol assimilation is supposed to involve pentose phosphate reactions, but the details are not fully clarified to date.

While there are several studies analyzing cellular reactions of *P. pastoris* to methanol induction in context of recombinant protein production [14–18], the response of non-recombinant strains to the different carbon sources is largely unknown. Thus, we decided to investigate the cellular responses of *P. pastoris* cells not producing a recombinant protein to methanol and glucose, respectively, which are the two most widely used substrates for cultivation. To enable the same chemostat-controlled constant specific growth rates for direct comparability the methanol cultures were co-fed with glycerol. Availability of whole genome sequences made a number of transcriptome regulation studies of *P. pastoris*, analyzing the implications of growth rate [19], unfolded protein response (UPR) induction [20], oxygen availability [21], osmotic stress [22], or heterologous protein production [16, 23], become feasible. Analyses of the host proteome gave further insights into characteristics of *P. pastoris* grown at different temperatures [24], osmolarity [22], UPR induction [25], and oxygen supply [21]. More recently, *P. pastoris* strains producing an insulin precursor were analyzed for changes in the cellular proteome as adaptation response to methanol induction during fed batch cultivation using 2D-DIGE and subsequent mass spectrometry identification of differentially abundant proteins. High abundance of enzymes from the dissimilatory methanol metabolism and induction of the UPR were observed [14]. Regulation of cellular enzyme concentrations will cause changes in metabolic fluxes, eventually also leading to changes in free metabolite concentrations. Quantitative determination of intracellular fluxes is the key to a better understanding of metabolic networks. First genome-scale metabolic network models of *P. pastoris* [26, 27] and flux distributions of central carbon metabolism [28–30] indicate growth rate-related methanol (co-)assimilation with proposed implications for the pentose phosphate pathway [31].

The work at hand incorporates transcriptomics, proteomics, metabolomics, and fluxomics analyses of non-producing *P. pastoris* in steady-state cultures at a uniform specific growth rate comprising the carbon source as the investigated variable. This integrated systems level analysis allowed to reveal cellular processes that are co-regulated with methanol metabolism, such as vitamin biosynthesis and amino acid metabolism. Furthermore, these co-regulation patterns were the pre-requisite to elucidate the thus far unidentified steps of sugar phosphate rearrangements recycling xylulose-5-phosphate for methanol fixation. We propose, herein, a new model for the assimilation of methanol as a separate strictly regulated pathway, originating from duplication of the involved genes.

## Results and discussion

### Growth parameters of *P. pastoris* differ significantly on different substrates

*P. pastoris* CBS7435 was cultivated in chemostat cultivations at a fixed specific growth rate of 0.1 h^−1^, corresponding to approximately 60 % of μ_max_ on glucose [19]. Constant growth is a prerequisite to avoid growth rate-dependent effects during genome-scale analyses. As the maximum specific growth rate on pure methanol as a carbon source would be significantly lower, and intracellular carbon fluxes could not be analyzed on methanol alone, a mixed feed strategy applying glycerol-methanol co-feeding was employed. A methanol-glycerol mix of 8.5 g/L methanol and 49.0 g/L glycerol was employed based on experiments with *P. pastoris* proving that total methanol utilization and full induction of the methylotrophic pathway were realized under these conditions. Chemostats were run in three biological replicates per condition and samples for transcriptomics, proteomics, and metabolite analyses were taken in steady state after seven residence times as described in the Methods section. For metabolic flux analysis, separate chemostat cultivations employing ^13^C-labelled substrates were performed. Substrate limitation of all cultures, i.e. no residual glucose or methanol/glycerol, respectively, was confirmed by HPLC. The growth parameters derived from these cultures are summarized in Table 1. The CO_2_ exchange rate of cells grown on methanol/glycerol was 13 % lower compared to those grown on glucose while their oxygen uptake rate was 30 % higher. The higher oxygen uptake rate of methanol/glycerol-grown *P. pastoris* can be explained by the higher degree of reduction of methanol and glycerol compared to glucose. As methanol oxidation to formaldehyde by AOX is an exothermic oxygen consuming reaction, an equimolar amount of oxygen is needed only to pass methanol into cellular metabolism. The biomass yield was slightly higher for cells grown on methanol/glycerol compared to glucose, which is in good agreement with data from the literature [28, 30]. Transcriptional regulation was analyzed using *P. pastoris*-specific DNA microarrays [20, 32], liquid chromatography-tandem mass spectrometry (LC/MS-MS) was used for differential proteomics and quantification of metabolites. Additionally, distribution of specific lipid classes were analyzed. Flux ratios were calculated from ^13^C labelling patterns in proteinogenic amino acids. The numerical results of these genome scale analyses can be found in Additional file 1.

**Table 1:** Growth parameters of *P. pastoris* grown on methanol/glycerol and glucose in chemostats at μ = 0.1 h^−1^

	Glucose	Glycerol	Methanol	CER	OUR	Biomass	Y_X/S_
	[mmol/(gCDW*h)]	[mmol/(gCDW*h)]	[mmol/(gCDW*h)]	[mmol/(gCDW*h)]	[mmol/(gCDW*h)]	[g/L]	[gCDW/gSubstrate]
Glucose	1.02 ± 0.03	–	–	2.11 ± 0.07	2.39 ± 0.07	28.1 ± 0.3	0.54 ± 0.01
Glycerol/Methanol	–	1.64 ± 0.06	0.81 ± 0.04	1.86 ± 0.05	3.09 ± 0.08	31.6 ± 0.3	0.57 ± 0.02

*CDW* Cell dry weight, *CER* CO_2_ exchange rate, *OUR* Oxygen uptake rate, *Y*_*XS*_ Biomass yield

### Transcriptome and proteome are significantly co-regulated

**Fig. 1:**
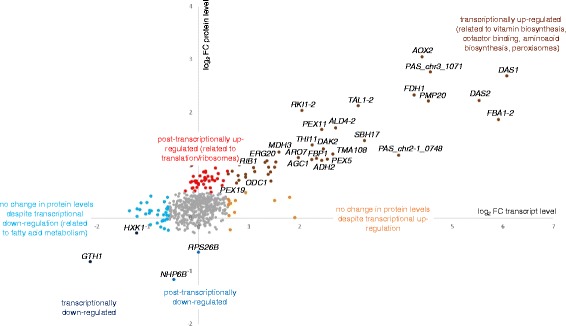
Co-regulation of transcripts and proteins of *P. pastoris* grown on methanol/glycerol compared to glucose in chemostats at μ = 0.1 h^−1^. Log_2_ fold changes of transcripts versus log_2_ fold changes of proteins for the 575 data pairs are shown which have been grouped based on their regulation patterns. Significantly enriched GO terms in these groups are indicated. Criteria for up/down-regulation of transcript and protein levels are described in the Methods section

At the transcriptional level, 406 of 5,354 genes were significantly differentially expressed on methanol/glycerol and glucose. As protein abundance, however, does not necessarily directly correlate with transcription [33], we also measured differential proteome regulation using 2D-LC-MS of Tandem Mass Tag labelled total protein samples and obtained quantitative data for 575 cellular proteins. In agreement with the literature [33–35], where a positive correlation between protein concentration and the abundance of the transcript has been described, we could mainly quantify proteins with higher transcript levels. Figure 1 shows 575 data pairs with mean log_2_ fold changes in transcript and protein levels of the methanol/glycerol experiments compared to the glucose experiments. Proteins and their transcripts were significantly co-regulated (r = 0.78, r^2^ = 0.61). Lu et al*.* [34] have shown that, in *Saccharomyces cerevisiae*, protein levels are determined to 73 % by transcription. Similarly, we observed that transcriptional control determined the regulation of protein abundance by 61 %.

Of the 575 genes with available transcriptomics and proteomics data, 130 (23 %) were differentially regulated at the transcript and/or protein level, the largest group being up-regulated at both levels. Based on the differential changes at both protein and transcript levels, data have been allocated to seven groups and analyzed for overrepresentation of functional groups (Fig. 1 and Additional file 1). As expected, during growth on methanol/glycerol there are strongly increased levels of transcripts and proteins involved in methanol metabolism and peroxisome formation, while two proteins needed mainly on glucose, hexokinase and high affinity glucose transporter, had lower abundance on both transcript and protein level. Higher levels of proteins of the translation machinery and cytoskeleton organization in cells grown on methanol/glycerol were not met by higher transcript levels, thus indicating a post-transcriptional regulation while the significant down-regulation of transcripts for lower glycolysis and fatty acid beta-oxidation was not reflected in protein levels (Fig. 1). These processes will be described in more detail below.

To confirm that the gene regulations attributed to methanol cultivation in this work are truly due to methanol, and not to glycerol as a co-substrate, we compared these regulation patterns to transcript regulation obtained in fed batch cultivations using methanol, glycerol, or glucose (Additional file 2). Thereby, we could confirm that all genes discussed to be regulated by methanol utilization in the present study are actually induced by methanol.

### Peroxisome proliferation is strongly up-regulated on methanol

Methanol-induced cells show an up-regulation in genes encoding proteins essential for peroxisome biogenesis and proliferation. Fourteen *PEX*-genes were markedly up-regulated on the transcriptome level, three of them also on the proteome level (Additional file 1). Genes of the peroxisomal import machinery encoding the docking complex (*PEX13*, *PEX14*, *PEX17*) and RING-finger complex (*PEX2*, *PEX10*, *PEX12*) are up-regulated at almost equal levels. Among peroxins required for the import of peroxisomal matrix proteins [36], *PEX11* and its isoform *PEX11C* were amongst the highest up-regulated genes (3- to 6-fold higher levels), while members of the Pex23-family were not induced. This distinction seems to be specific for *P. pastoris*, as both gene groups were up-regulated upon methanol induction in *H. polymorpha* [10]. A similar regulation of *P. pastoris PEX* genes has also been described recently by Prielhofer et al*.* [37], who observed that genes encoding the Pex7/Pex20-mediated import machinery and the Pex23-family were only up-regulated in conditions inducing expression of beta-oxidation genes but not upon methanol induction. Most key players of the peroxisomal methanol utilization pathway, such as AOX, catalase, and DAS, rely on the Pex5-mediated PTS1 import pathway [9]; thus, up-regulation of receptors, which recognize a peroxisomal targeting signal sequence (PTS), was restricted to PTS1-specific *PEX5* [38]. Conversely, *PEX7*, encoding a signal receptor for the signal sequence PTS2, was slightly down regulated. This finding is in agreement with previous studies [39, 40] demonstrating that *H. polymorpha* and *P. pastoris* do not require the PTS2 import pathway for growth on methanol. Unchanged expression levels of Pex7 and Pex20 encoding its accessory protein also correlated with unaltered protein levels of beta-oxidation enzymes. Genes encoding auxiliary functions in matrix protein import and quality control were induced: the putative peroxisomal Lon-protease Pim1-2 and the peroxisomal ATP importer Pmp47 (PAS_chr3_0099) required for import of DAS were strongly up-regulated (log_2_FC +3.78). Further, up-regulation at both transcriptome and protein level was detected for the glutathione peroxidase Pmp20, a peroxisomal protein which might also be involved in the detoxification of H_2_O_2_ in the peroxisome of methanol growing cells.

### The xylulose-monophosphate cycle of methanol assimilation utilizes a duplicated methanol inducible enzyme set and is entirely localized to peroxisomes

The key players of the methanol utilization pathway have been identified during the last 30 years [12, 13]; however, major steps of the assimilation pathway still remain to be resolved. Briefly, methanol is oxidized to formaldehyde by AOX (Aox1 and Aox2 in *P. pastoris*) within the peroxisomes, thereby generating stoichiometric amounts of H_2_O_2_. Formaldehyde is further converted in two possible routes, either dissimilatory by glutathione-dependent formaldehyde dehydrogenase, S-formyl glutathione hydrolase, and formate dehydrogenase yielding NADH and CO_2_, or assimilatory by the action of DAS (Das1 and Das2 in *P. pastoris*). DAS catalyzes the fusion of formaldehyde to xylulose-5-phosphate (XYL5P), thereby generating dihydroxyacetone and glyceraldehyde-3-phosphate (GAP). These intermediates are further converted by dihydroxyacetone kinase (DAK), fructose-1,6-bisphosphate aldolase, and fructose-1,6-bisphosphatase, to finally yield one molecule of GAP per three molecules of methanol, which is then used for the generation of biomass and energy. It is generally assumed that XYL5P gets recycled through rearrangements in the pentose phosphate pathway (PPP), although the detailed mechanism of these rearrangements as well as the interplay of PPP and peroxisomes is still unknown.

All known enzymes of the methanol utilization pathway have significantly higher transcript and protein levels when methanol is present (Fig. 1, upper right quadrant). Interestingly, our analysis revealed that *P. pastoris* does not only have a second isoform of fructose-1,6-bisphosphate aldolase (designated as Fba1-2) as reported by Küberl et al*.* [41], but also isoforms of the PPP enzymes transaldolase (Tal1-2), ribose-5-phosphate ketol-isomerase (Rki1-2), and ribulose-5-phosphate 3-epimerase (Rpe1-2). All these isoforms were found among the group of up-regulated gene-protein pairs, except for Rpe1-2 which was not identified at the proteomic level. Sequence analysis predicted that Fba1-2, Tal1-2, Rki1-2, and Rpe1-2 each contain a PTS1 peroxisomal targeting signal [42, 43], indicating their potential involvement in a separate peroxisomal methanol assimilation pathway. On the contrary, their respective cytosolic or mitochondrial isoforms (Fba1-1, Tal1-1, Rki1-1, and Rpe1-1) were not differentially regulated and do not contain a peroxisomal targeting sequence. The same regulation pattern was observed comparing cultures grown on methanol alone to those grown on glycerol or glucose (Additional file 2).

Subsequently, cellular fractions enriched of highly pure peroxisomes were isolated from methanol- or glucose-grown *P. pastoris* according to the protocol established by Wriessnegger et al*.* [44], and subjected to proteomics analyses. In this way, we demonstrated that all relevant enzymes for methanol assimilation were present only in methanol-derived peroxisomal fractions, but not in glucose-derived peroxisome fractions (Table 2). The relative enrichment of proteins in the peroxisomal fractions compared to total cell homogenates was quantified as average weighted ratios of the peak areas of respective peptides. This was consequently only possible with methanol-derived samples where peptides of the proteins of interest had been identified. Table 3 shows MASCOT scores as indicators of identification, and the average ratios of protein abundance in peroxisomal vs. homogenate samples, normalized to Aox1. The methanol assimilation pathway enzymes discussed above were enriched at the same level or higher than Aox1 in the peroxisomal fractions, just as several selected peroxisomal proteins, while cytosolic proteins of glycolysis, PPP, and methanol dissimilation were either not identified at all or markedly depleted in peroxisomal preparations. Only DAK of the methanol assimilation pathway was rather depleted compared to Aox1. Luers et al. [45] described that DAK localizes to the cytosol despite having a PTS1 signal. We could, however, quantify DAK also in peroxisomal fractions of methanol-grown cells, indicating that this enzyme can localize in more than one compartment.

**Table 2:** Transcriptional and post-transcriptional regulation of genes related to the methanol metabolism, the pentose phosphate pathway, and the glyoxylate cycle. Presence of the corresponding protein in the peroxisomal fraction (of methanol- or glucose-grown *P. pastoris*) is indicated as well as prediction of peroxisomal targeting based on the C-terminal amino residues of the proteins using the PTS1 predictor [43]

Pathway	Short name ^a^	ORF name ^b^	Description	Transcript (methanol/glycerol vs glucose) ^c^	Protein (methanol/glycerol vs glucose) ^d^	Presence in the peroxisome fraction (methanol) ^e^	Presence in the peroxisome fraction (glucose) ^e^	Prediction of peroxisomal targeting ^f^	Last 12 C-terminal amino acid residues
Methanol assimilation	AOX1	PP7435_Chr4-0130/ PAS_chr4_0821	Alcohol oxidase	up	n.i.	yes	no	yes	LGTYEKTGLARF
	AOX2	PP7435_Chr4-0863/ PAS_chr4_0152	Alcohol oxidase	up	up	n.i.	n.i.	yes	LGTYEKTGLARF
	DAS1	PP7435_Chr3-0352/ PAS_chr3_0832	Dihydroxyacetone synthase variant 1	up	up	yes	no	no	HDLKGKPKHDKL
	DAS2	PP7435_Chr3-0350/ PAS_chr3_0834	Dihydroxyacetone synthase variant 2	up	up	yes	no	no	TDLKGKPKHDKL
	DAK2	PP7435_Chr3-0343/ PAS_chr3_0841	Dihydroxyacetone kinase	up	up	yes	no	Twilight zone	ITDAYFKSETKL
	FBA1-2	PP7435_Chr1-0639/ PAS_chr1-1_0319	Fructose-1,6-bisphosphate aldolase	up	up	yes	no	yes	HAAGTFKSESKL
	FBP1	PP7435_Chr3-0309/ PAS_chr3_0868	Fructose-1,6-bisphosphatase	up	up	yes	no	no	LTKKIKIQSVNL
	SHB17	PP7435_Chr2-0185/ PAS_chr2-2_0177	Sedoheptulose-1,7-bisphosphatase	up	up	yes	no	no	VVPVEEAEADRA
	RKI1-2	PAS_chr4_0212	Ribose-5-phosphate ketol-isomerase	up	up	yes	no	yes	ITSLSVSVPARL
	TAL1-2	PAS_chr2-2_0338	Transaldolase	up	up	yes	no	yes	VPSLFRRVLSKL
	RPE1-2	PP7435_Chr3-0772	D-ribulose-5-phosphate 3-epimerase	up	n.i.	n.i.	n.i.	Twilight zone	QKKAKAKPKPNL
Peroxisomal protein	CTA1	PP7435_Chr2-0137/ PAS_chr2-2_0131	Catalase A	up	n.q.	yes	no	yes	QLSPRGDSAARL
	PMP20	PP7435_Chr1-1351/ PAS_chr1-4_0547	Peroxiredoxin	up	up	yes	no	yes	KHSSADRVLAKL
Methanol dissimilation	FLD	PP7435_Chr3-0140/ PAS_chr3_1028	Bifunctional alcohol dehydrogenase and formaldehyde dehydrogenase	up	n.q.	no	no	no	AGNCIRAVITMH
	FGH1	PP7435_Chr3-0312/ PAS_chr3_0867	Esterase that can function as an S-formylglutathione hydrolase	up	n.q.	no	no	no	HAAHHAKYLGLN
	FDH1	PP7435_Chr3-0238/ PAS_chr3_0932	NAD(+)-dependent formate dehydrogenase	up	up	yes	no	no	KTKAYGNDKKVA
Pentose phosphate pathway oxidative branch	ZWF1	PP7435_Chr2-0993/ PAS_chr2-1_0308	Glucose-6-phosphate dehydrogenase	not changed	not changed	no	no	no	WPVTRPDVLHKM
	SOL3	PP7435_Chr3-0037/ PAS_chr3_1126	6-phosphogluconolactonase	not changed	n.q.	no	no	Twilight zone	ALSGVSVSTSKY
	GND2	PP7435_Chr3-0944/ PAS_chr3_0277	6-phosphogluconate dehydrogenase	not changed	not changed	yes	yes	no	KGGNVSASTYDA
Pentose phosphate pathway non-oxidative branch	RPE1-1	PP7435_Chr3-0771	D-ribulose-5-phosphate 3-epimerase	n.a.	up	n.i.	n.i.	no	QDSLKKKGLLDE
	RKI1-1	PAS_chr4_0213	Ribose-5-phosphate ketol-isomerase	up	n.q.	n.i.	n.i.	no	GNEDGSVATLTL
	TKL1	PP7435_Chr1-0919/ PAS_chr1-4_0150	Transketolase	not changed	not changed	no	no	no	SPLNKAFESVHA
	TAL1-1	PP7435_Chr2-0357/ PAS_chr2-2_0337	Transaldolase	not changed	not changed	yes	yes	no	TLLNLLKEKVQA
Glyoxylate cycle	CIT1	PP7435_Chr1-0426/ PAS_chr1-1_0475	Citrate synthase	not changed	n.q.	yes	no	no	EKYIELVKGLGK
	ACO1	PP7435_Chr1-0105/ PAS_chr1-3_0104	Aconitase	not changed	not changed	yes	no	no	ALNNMAAVKASK
	ACO2	PP7435_Chr3-0541/ PAS_chr3_0659	Aconitase	not changed	n.q.	no	no	no	INYIGRLKREQQ
	ICL1	PP7435_Chr1-1123/ PAS_chr1-4_0338	Isocitrate lyase	not changed	n.q.	no	no	no	GAGVTEDQFKDH
	MLS1	PP7435_Chr4-0820/ PAS_chr4_0191	Malate synthase	not changed	up	n.i.	n.i.	no	LESSPVDLDSLK
	MDH3	PP7435_Chr4-0136/ PAS_chr4_0815	Peroxisomal malate dehydrogenase	up	up	yes	no	no	NIAKGTAFIAGN
	MLS2	PP7435_Chr1-1255/ PAS_chr1-4_0459	Malate synthase	up	n.i.	n.i.	n.i.	Twilight zone	STIPINIHQQKL
	AAT1	PP7435_Chr1-0511/ PAS_chr1-1_0200	Aspartate aminotransferase	up	n.q.	yes	no	no	YLANAIHEVTTN
	AAT2	PAS_chr4_0974	Aspartate aminotransferase	not changed	n.q.	yes	no	no	RVAAAIDQVVRV
	ODC1	PP7435_Chr3-1205/ PAS_chr3_0040	Oxoglutarate-malate shuttle	up	up	yes	no	no	FTTCMDFFRTLQ
	OSM1	PP7435_Chr3-1001/ PAS_chr3_0225	Fumarate reductase	up	n.i.	n.i.	n.i.	Twilight zone	YLLKSLSNYHKL

^a^In some cases, *P. pastoris* has two homologs of the same *S. cerevisiae* gene (i.e. TAL1-1 and TAL1-2)

^b^ORF names of two *P. pastoris* strains: *P. pastoris* CBS7435/*P. pastoris* GS115 (the sequences are identical in the two strains; however, in a few cases only the ORF name of one strain is reported because the sequence of the other strain is not or wrongly annotated.)

^c^ *n.a.* not available on microarray

^d^ *n.i. * not identified; *n.q. * identified but could not be quantified

^e^ *n.i. * not identified in the peroxisome fraction

^f^ Prediction of peroxisomal targeting with PTS1 predictor [43] (classification according to [42]: yes: predicted; twilight zone: questionable but with reasonable estimated false-positive rate; no: not predicted)

**Table 3:** Identification and quantification of methanol metabolic enzymes and control proteins in peroxisomal preparations (Pex) and homogenates (Hom) of *P. pastoris* grown on methanol. MASCOT scores indicate identification of the respective proteins in the samples while peak areas of the identified peptides were used for quantification. To normalize the dataset, average ratios of the summarized peak areas of Aox1 peptides of peroxisomal samples vs homogenates were set to 1, and all ratios were calculated in relation to this. Peroxisomal proteins serve as positive control, while methanol dissimilation, pentose phosphate pathway (PPP), and glycolysis-related enzymes are negative controls localized to the cytosol

Short name	Function/localization	Description	MASCOT Score Pex1	MASCOT Score Pex2	MASCOT Score Hom1	MASCOT Score Hom2	ratio peak area Pex/Hom
AOX1	Methanol assimilation	Alcohol oxidase 1	1542.8	1157.2	1021.4	1061	1.00
DAS1	Methanol assimilation	Dihydroxyacetone synthase 1	1918.9	1503	971.2	970.4	14.79
DAS2	Methanol assimilation	Dihydroxyacetone synthase 2	1797.9	1473.2	986.3	961.1	7.10
DAK2	Methanol assimilation	Dihydroxyacetone kinase	226.9	0	666.2	550.4	0.26
FBA1-2	Methanol assimilation	Fructose-bisphosphate aldolase	464.8	185.7	287	291.5	0.96
FBP1	Methanol assimilation	Fructose-1,6-bisphosphatase	623.8	419.8	585	576.8	2.42
SHB17	Methanol assimilation	Sedoheptulose-1,7-bisphosphatase	357.4	314	191	145.2	3.05
RKI1-2	Methanol assimilation	Ribose-5-phosphate ketol-isomerase	217.1	0	0	139.8	2.97
TAL1-2	Methanol assimilation	Transaldolase	374.3	279.8	0	0	> > 1
CTA1	Peroxisomal protein	Catalase	907.3	434.4	414.3	304.3	1.26
PEX3	Peroxisomal protein	Peroxisomal biogenesis factor	119.2	109.9	0	0	> > 1
PEX5	Peroxisomal protein	Peroxisomal targeting signal 1 receptor	86.1	62.5	127.8	36.3	1.09
PEX11	Peroxisomal protein	Peroxisomal membrane protein	523.4	262.1	221.5	151.8	4.26
PEX14	Peroxisomal protein	Peroxisomal membrane protein	145.2	103.7	0	0	> > 1
PMP20	Peroxisomal protein	Peroxiredoxin	536	437.3	260.7	274.2	1.49
PMP47	Peroxisomal protein	Peroxisomal membrane protein	539.5	345.7	165.9	162.5	8.02
FLD	Methanol dissimilation	Formaldehyde dehydrogenase	0	0	577.9	424.4	0.00
FGH1	Methanol dissimilation	S-formylglutathione hydrolase	0	0	455.4	432.4	0.00
FDH1	Methanol dissimilation	Formate dehydrogenase	491.1	303.5	910.5	856.7	0.12
TAL1-1	PPP	Transaldolase	220.2	0	376.7	206.5	0.38
TKL1	PPP	Transketolase	0	0	85	166	0.00
ZWF1	PPP	Glucose-6-phosphate 1-dehydrogenase	0	0	0	46.7	0.00
GND2	PPP	6-phosphogluconate dehydrogenase	247.1	66.6	626.8	522.9	0.26
FBA1-1	Glycolysis	Fructose-bisphosphate aldolase	0	0	347.3	379.1	0.00
HXK1	Glycolysis	Hexokinase	0	0	100.6	195.6	0.00
TDH3	Glycolysis	Glyceraldehyde-3-phosphate dehydrogenase	0	0	629.6	705.5	0.00
PGK1	Glycolysis	Phosphoglycerate kinase	0	0	372.1	224.3	0.00
GPM1	Glycolysis	Phosphoglycerate mutase	0	0	121.7	108.4	0.00

**Fig. 2:**
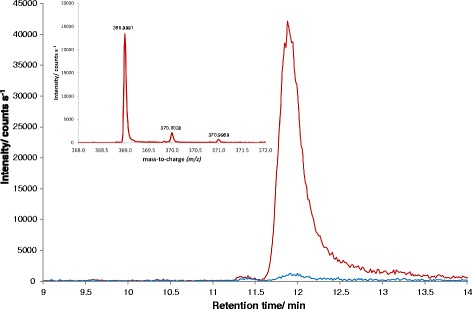
Extracted ion chromatogram (*m/z* = 368.9993 ± 10 ppm) of a sample grown on methanol (red) or glucose (blue) showing the sedoheptulose-1,7-bisphosphate peak at a retention time of 12 min. In the upper left corner the mass spectrum (*m/z*) of the peak of the methanol-grown sample after background subtraction is shown. The difference between the measured accurate mass and the calculated exact mass is 0.53 ppm (0.2 mDa)

Additionally, one of the unidentified ORFs present among the up-regulated gene/protein pairs was identified to be the homolog of *S. cerevisiae*, YKR043C, which was recently reported to encode sedoheptulose-1,7-bisphosphatase (Shb17) [46]. Further, this protein was found to be enriched in the peroxisomes in methanol-grown *P. pastoris* (Table 2). Shb17 was shown to hydrolyze sedoheptulose-1,7-bisphosphate (S1,7BP) to sedoheptulose-7-phosphate in a thermodynamically driven pathway for the synthesis of pentose-5-phosphates alternative to PPP [46]. S1,7BP was not among the quantified metabolites in our initial metabolomics analyses due to the lack of a commercially available standard. After receiving purified S1,7BP from Amy Caudy (University of Toronto, CA), a previously unidentified substance with differential abundance could be unambiguously assigned as S1,7BP. The signal-to-noise ratios of all samples with glucose-treatment were below 5. In the methanol-grown samples, the signal-to-noise ratios were 84, 111, and 116, clearly indicating the presence of S1,7BP in methanol-grown *P. pastoris* cells, contrary to glucose-grown cells (Fig. 2). This result prompted us to reconsider the pentose phosphate rearrangements leading to the formation of XYL5P for methanol assimilation.

### Methanol assimilation employs an alternative xylulose-5-phosphate forming pathway via sedoheptulose-1,7-bisphosphate

**Fig. 3:**
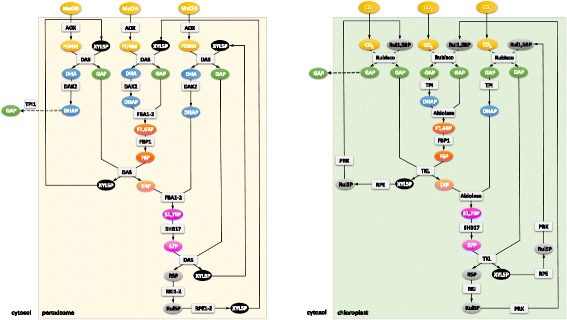
Regeneration of pentose phosphates. Left: Methanol assimilation through the xylulose-monophosphate cycle: proposed rearrangements employing an alternative XYL5P forming pathway via S1,7BP. The net reaction of methanol assimilation is the formation of one GAP molecule from three methanol molecules. Right: Rearrangement reactions of the Calvin cycle. Regeneration of ribulose-1,5-bisphosphate (Rul-1,5-BP) needed for CO_2_ fixation in chloroplasts of plants via S1,7BP. For simplicity, the initial reaction steps after carbon fixation are condensed. The enzyme RuBisCO catalyzes the fixation of CO_2_ to Rul-1,5-BP, which yields two 3-phosphoglycerate molecules, which are phosphorylated to 1,3-bisphosphoglycerate by phosphoglycerate kinase, and then reduced to GAP by glyceraldehyde 3-phosphate dehydrogenase. Involved metabolites are in oval signs, genes/proteins are shown in rectangular signs. The colors of the individual metabolites serve for better readability of the figure, that is, chemically related compounds share the same color. The regulation pattern and the cellular localization of the proteins is given in Table 2

While it would be stoichiometrically possible that XYL5P is regenerated through the canonical non-oxidative branch of the pentose phosphate pathway, our genomic, transcriptomic, and proteomic data point to another direction of C1 assimilation. It appears most likely that *P. pastoris* and other methylotrophic yeasts evolved a specialized set of enzymes for sugar phosphate rearrangements which is specifically induced by growth on methanol and localizes to peroxisomes. Figure 3 shows our proposed pathway for the rearrangement reactions, with 1 GAP molecule per 3 molecules of methanol as the net result. Thereby, F6P (generated from GAP and dihydroxyacetone phosphate (DHAP) by the action of Fba1 and fructose-1,6-bisphosphatase) and another GAP are interconverted to erythrose-4-phopsphate and XYL5P in a transketolase reaction. Erythrose-4-phopsphate is then condensed with DHAP to form S1,7BP, a reaction shown to be catalyzed by the aldolase Fba1 in yeast and plants [46]. We propose that peroxisomal Fba1-2 or Tal1-2 might be the responsible enzyme for this reaction in *P. pastoris*. Shb17 catalyzes the dephosphorylation of S1,7BP to sedoheptulose-7-phosphate, which is finally converted to two XYL5P by transketolase, Rki1-2, and Rpe1-2. As *P. pastoris* Tkl1 is cytosolic and not induced in the presence of methanol, we propose that Das1 and/or Das2, both homologs of Tkl1, catalyze this reaction. Overall, in a process driven by the net loss of one high-energy phosphate bond, Sbh17, together with transaldolase and transketolase, convert five moles of triose-phosphate into the three moles of XYL5P required for fixation of three moles of formaldehyde by DAS. Localization of this entire pathway in the same compartment makes import of XYL5P into peroxisomes obsolete, which was proposed to be necessary by Douma et al*.* [47] according to the classical model of methanol assimilation. Thus, the net peroxisomal flux of carbon would be one mole DHAP or GAP out of peroxisomes per three moles of methanol.

Based on our data, we propose here a novel carbon assimilation pathway (Fig. 3, left) that shares the concept of compartmentalization with plants [48] and cyanobacteria [49]. According to this model, DAS is responsible for C-C bond formation similar to the mechanism of RuBisCO, followed by a cyclic pathway (the equivalent to the Calvin cycle; Fig. 3, right) for regeneration of the pentose phosphate substrate of the carboxylation reaction. Shb17 has been shown to drive the flux from erythrose-4-phosphate and DHAP toward ribose-5-phosphate during riboneogenesis in *S. cerevisiae* in a reaction similar to the Calvin cycle [46]. A similar mechanism driving the flux towards XYL5P is proposed here in methanol-induced *P. pastoris* and probably also in other methylotrophic yeasts. Supporting this hypothesis we also found PTS1 containing isoforms of Fba1 and Tal1 in the *H. polymorpha* genome sequence by BLAST analysis.

Tandem gene duplication occurs with high frequency and has been reported to be a major contributor of new genetic material [50]. Several models for the occurrence of gene duplications have been proposed (reviewed in [50, 51]). Among them unequal crossing over can lead to tandem duplication, as it is observed here. Duplicated genes have a high probability of being lost again unless they acquire a new function [50]. Byun-McKay and Geeta [52] have proposed that subcellular relocalization of duplicate gene products may play an important role in stabilizing duplications and acquiring new functions. While they extend their idea only to N-terminal mutations modifying targeting sequences to the endoplasmic reticulum, mitochondria, or chloroplasts, it may well be that C-terminal mutations may have enabled peroxisomal relocation of duplicate gene products in an ancestor of methylotrophic yeasts. One may envisage that compartmentalized xylulose-5-monophosphate pathway enzymes would constitute novel functions which underwent positive selection, leading to a highly regulated peroxisomal pathway as observed herein, while leaving the PPP unaffected.

### The central carbon metabolism is reverted to gluconeogenesis

**Fig. 4:**
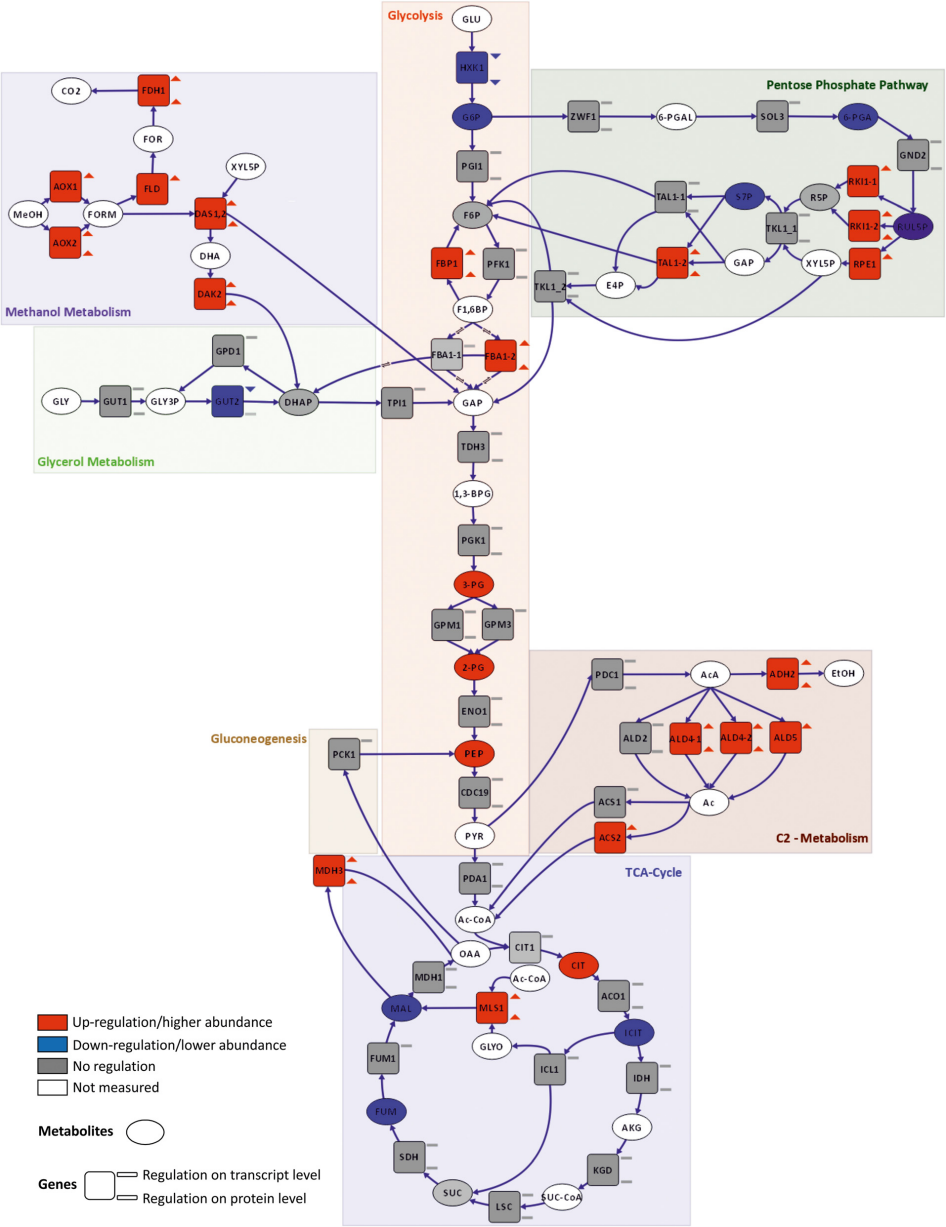
Differential regulation of central carbon metabolism comparing methanol/glycerol- and glucose-grown cells. Visualization of changes in transcript (square, upper symbol), protein (square, lower symbol), and metabolite (oval) levels. Red: up-regulation on methanol/glycerol; blue: down-regulation on methanol/glycerol; gray: not differentially regulated; white/no symbol: not measured. Criteria for up-/down-regulation of transcript, protein, and metabolite levels are described in the Methods section

**Fig. 5:**
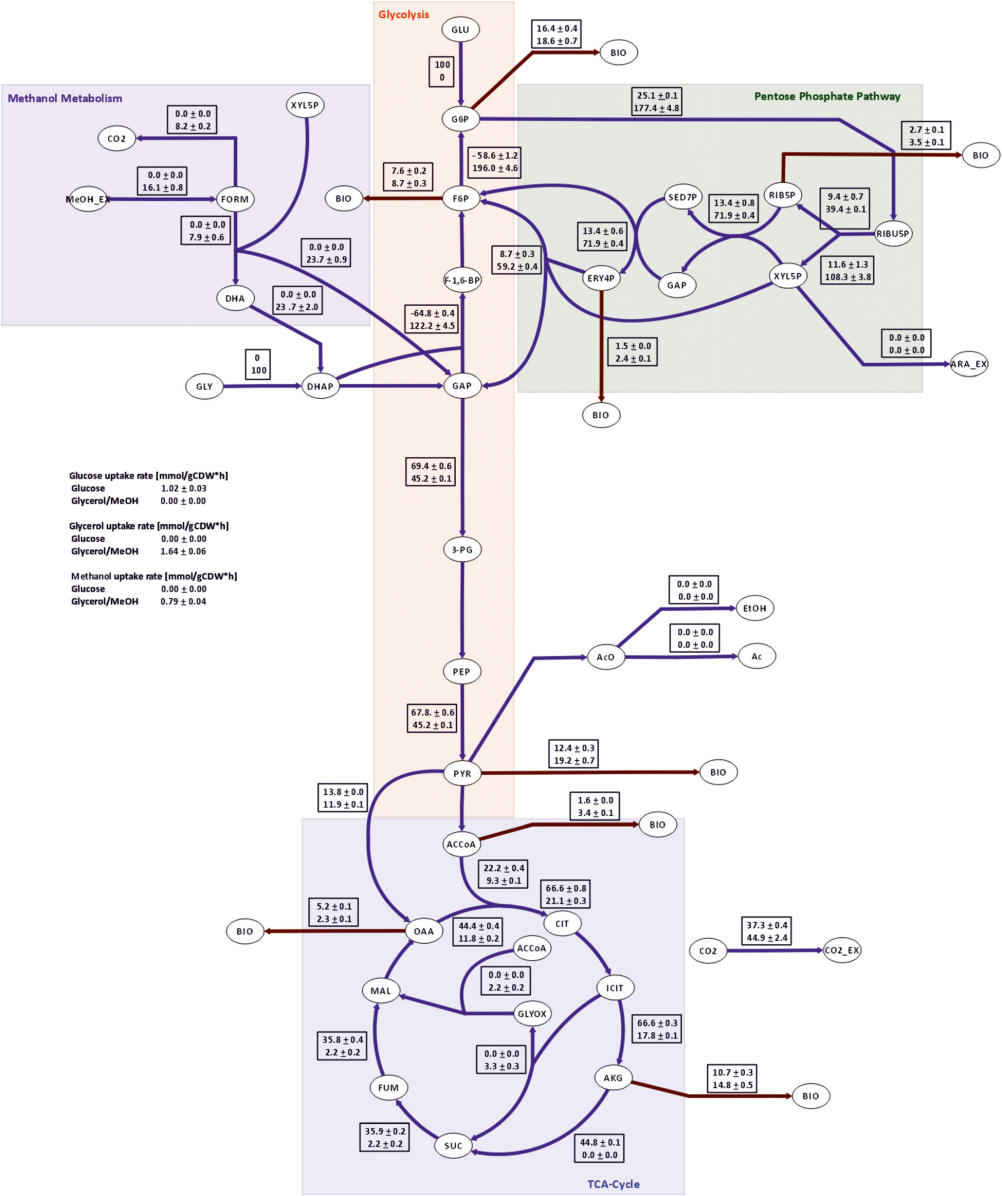
Intracellular metabolic flux distributions of methanol/glycerol- and glucose-grown cells. The flux values are normalized to glycerol or glucose uptake, respectively, and presented in [% Cmol]. The upper value in the rectangular boxes represents the flux distribution on glucose and the lower value the flux distribution on methanol/glycerol. For reversible reactions only the net fluxes are presented

Growth on non-carbohydrate carbon sources necessitates the synthesis of hexoses and pentoses for the biosynthesis of macromolecules, which is accomplished by reverting the carbon flux to gluconeogenesis. Glycolysis and gluconeogenesis share several enzymes, while the irreversible, highly exergonic steps of glycolysis are bypassed. Therefore, exactly these reactions are the control steps of flux direction, and their regulation indicates the activity of gluconeogenesis. Both methanol and glycerol enter the central carbon metabolism at the level of C3-molecules (DHAP and GAP). One of the key regulatory enzymes of the upper part of glycolysis/gluconeogenesis is fructose-1,6-bisphosphatase, which we found to be up-regulated in methanol/glycerol-grown cells at the transcriptomic and proteomic level (Fig. 4). The other key regulatory enzymes of the lower part, pyruvate carboxylase and phosphoenolpyruvate carboxykinase, showed no differential regulation comparing both conditions, which is consistent with the fact that carbon flux from methanol and glycerol enters the central carbon metabolism at the point of glyceraldehyde-3-phosphate. Correspondingly, there was only a minor difference in the calculated fluxes from glyceraldehyde-3-phosphate towards pyruvate while the upper glycolytic flux was reverted on methanol/glycerol towards glucose-6-phosphate (Fig. 5). Additionally, the absence of extracellular glucose rendered low and high affinity glucose transporters *HXT1* and *GTH1* as well as hexokinase *HXK1* obsolete which all had significantly lower transcript (and protein) levels on methanol/glycerol (Fig. 1, lower left).

Flux through the lower branch of glycolysis was lower on methanol/glycerol (Fig. 5) which fits to the observed lower TCA-cycle flux and the accumulation of three glycolytic intermediates (2-phosphoglycerate, 3-phosphoglycerate, and phosphoenolpyruvate). Transcript and protein levels of most glycolytic enzymes did not change (Fig. 4). Similarly, in *S. cerevisiae*, a poor correlation between fluxes and transcript levels of genes of this pathway was observed [53]. The low TCA-cycle flux indicates that methanol dissimilation is a major source for NADH and energy production in cells grown on methanol/glycerol.

### Pentose phosphate pathway flux is increased on methanol/glycerol

The PPP serves for the generation of NADPH for reductive assimilatory processes and for the generation of ribose-5-phosphate as a precursor for nucleic acids. Other PPP intermediates are used as precursors for other metabolic pathways like synthesis of histidine, nucleotides, and riboflavin. Methanol assimilation, using sugar phosphate intermediates in a cyclic fashion, has to be regarded separately from PPP, as outlined above.

The high gluconeogenic flux on methanol/glycerol was accompanied by a high flux through the PPP (Fig. 5), enabling a high specific production rate of reduced NADPH of appr. 3 mmol g^−1^ h^−1^. About 10 % of this higher NADPH production is needed for amino acid synthesis for the higher protein content of cells grown on methanol/glycerol. A higher PPP flux also provides for more ribose for nucleotide and riboflavin synthesis (see below). Cytosolic pentose phosphate pathway genes, however, were not differentially regulated at the different media (Table 2), contrary to the methanol-induced peroxisomal isoforms suggested to be employed in methanol assimilation in this compartment.

### Up-regulation of the malate-aspartate shuttle serves for mitochondrial import of NADH generated by methanol dissimilation and correlates with decreased TCA-cycle flux in methanol/glycerol-grown *P. pastoris*

The TCA-cycle as a central hub for cellular metabolism is dedicated to energy production and supply of precursors for several other metabolic pathways. Again, the most striking C-source-dependent differences were observed on the flux level, being 3.2 times lower on methanol/glycerol, mainly being controlled by the lower influx of acetyl-CoA into the TCA-cycle (Fig. 5). The low TCA-cycle flux on methanol is mainly diverted to glutamate, thus contributing only marginally to energy production. No significant changes in transcript or protein levels of genes connected to the TCA-cycle were observed. Nevertheless, we found marked differences in TCA-cycle metabolites. Citrate levels in methanol/glycerol-grown cells were higher than in glucose-grown cells, whereas levels for isocitrate, fumarate, and malate were lower (Fig. 4). Taken together, these data indicate that, on methanol/glycerol, the TCA-cycle reactions are mainly employed for production of metabolic precursors for biomass formation rather than producing energy through the respiratory chain. Methanol utilization has a major impact on the energy state of the cells, as two moles of NADH are produced via dissimilation of one mole of methanol to CO_2_. Intracellular flux calculation showed that about half of the methanol was dissimilated to CO_2_ and therefore additional NADH was produced which may consequently lead to down-regulation of TCA-cycle flux.

Dissimilatory oxidation of formaldehyde takes place in the cytosol. Therefore, the produced NADH has to be transported via the inner mitochondrial membrane to drive the generation of ATP. For the transport of electrons via the mitochondrial membrane several shuttle systems exist, most importantly the malate-aspartate shuttle. The homologs of the malate-α-ketoglutarate transporter Odc1 [54] and the glutamate-aspartate transporter Agc1 [55] were both highly up-regulated at transcript and protein levels, indicating the relevance of this NADH shuttle for methylotrophic ATP generation in mitochondria.

### The glyoxylate cycle is active in methanol/glycerol-grown *P. pastoris* and mainly localizes to the peroxisomes

The glyoxylate cycle is necessary for the utilization of non-fermentable carbon sources because of its ability to convert acetyl-CoA into C4 compounds that can be used for ATP generation in the mitochondria [56]. Isocitrate lyase converts isocitrate to glyoxylate and succinate, the former intermediate is then condensed with acetyl-CoA to form malate by malate synthase. Additionally, malate dehydrogenase, citrate synthase, and aconitase are required. This process is assumed to take place in the peroxisomes in non-*Saccharomyces* yeasts [8]. Indeed, we found most of the enzymes to be present in the peroxisomal fraction in methanol-grown cells (Table 2), only isocitrate lyase was found solely on methanol but predominantly in the cytosolic fraction. Furthermore, methanol-grown cells had increased transcript and/or protein levels of both putative peroxisomal malate synthase (PAS_chr1-4_0459, which we named Mls2) as well as of the cytosolic malate synthase and malate dehydrogenase. In agreement with this data, we also found increased glyoxylate cycle fluxes in cells grown on methanol/glycerol. We propose that the generated C4 compounds are mainly used as precursors for the biosynthesis of TCA-cycle-derived amino acids when using methanol/glycerol as substrate, rather than being shuttled to gluconeogenesis. In this line, no up-regulation of phosphoenolpyruvate carboxykinase, a key enzyme in lower gluconeogenesis, could be seen.

### Methanol-grown *P. pastoris* cells have a higher protein but lower free amino acid content

**Fig. 6:**
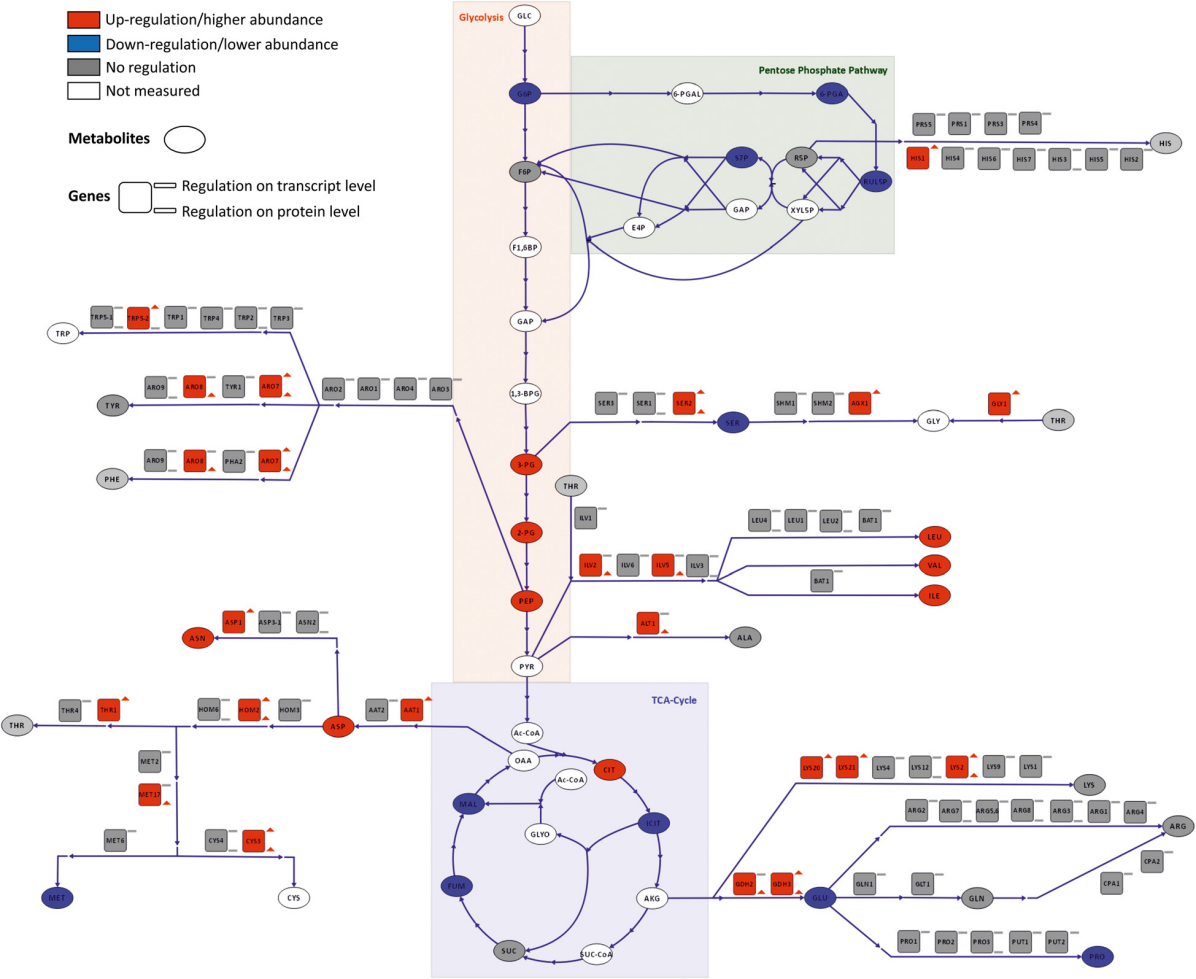
Differential regulation of amino acid synthesis pathways comparing methanol/glycerol- and glucose-grown cells. Visualization of changes in transcript, protein and metabolite levels. For an explanation of the symbols see legend to Fig. 4

Protein is the largest macromolecular component of cells, creating the highest demand for energy, reduction equivalents, and carbon flux. A change in substrate forces the cell to adapt, e.g. by varying the total protein content. Methanol/glycerol-grown cells had a 35 % higher protein content, with 0.54 g(protein) g(cell dry weight (CDW))^−1^ compared to glucose-grown cells with 0.40 g(protein) g(CDW)^−1^ (Table 4). Consequently, the levels of protein-bound amino acids were generally higher in cells grown on methanol/glycerol, while the level of free intracellular amino acids was approximately 20 % lower in methanol/glycerol-grown cells. The higher specific protein synthesis rate on methanol/glycerol (0.054 g(protein) g(CDW)^−1^ h^−1^ vs 0.040 g(protein) g(CDW)^−1^ h^−1^ on glucose) creates a higher drain of amino acids towards protein synthesis, which explains the generally low levels of intracellular free amino acids. The higher demand for amino acids creates a metabolic pull for the respective synthesis pathways. The higher demand for amino acids was well supported by the transcriptional and/or post-transcriptional up-regulation of genes involved in the biosynthesis of all twenty amino acids (Fig. 6). For histidine which derives from one intermediate of the pentose phosphate pathway, we saw regulation of *HIS1*, while for the amino acids which derive from glycolytic intermediates, regulation of Ser2 (serine), *AGX1* and *GLY1* (glycine), *TRP5-2* (tryptophan), Aro7 and Aro8 (tyrosine and phenylalanine), Ilv2 and Ilv5 (leucine, valine, isoleucine), and Alt1 (alanine) was observed. If we consider amino acids which derive from intermediates of the TCA-cycle, we saw regulation of *LYS20*, *LYS21* and Lys2 (lysine), Gdh2 and Gdh3 (glutamate), *AAT1* (aspartate), *ASP1* (asparagine), Hom2 (precursor of threonine, methionine, and cysteine), *THR1* (threonine), Met17 (precursor of methionine and cysteine), and Cys3 (cysteine).

**Table 4:** Composition of protein bound and free intracellular amino acids of *P. pastoris* grown on glucose or methanol/glycerol in chemostats at μ = 0.1 h^−1^

	Protein bound amino acids			Free intracellular amino acids			
	Methanol/glycerol	Glucose	Log_2_FC	Methanol/glycerol	Glucose	Log_2_FC	*P* value
	Average [mg/gCDW]	Average [mg/gCDW]		Average [mg/gCDW]	Average [mg/gCDW]		
Asx	48.8	30.2	0.69	5.42	2.91	0.89	0.00
Ala	32.1	20.2	0.67	0.90	1.02	−0.18	0.28
Arg	29.6	25.9	0.19	11.1	12.1	−0.12	0.47
Cys	5.59	4.56	0.30	–	–	–	–
Glx	87.0	68.0	0.36	22.3	29.4	−0.40	0.00
Gly	18.7	11.5	0.70	–	–	–	–
His	11.9	7.33	0.70	0.76	0.74	0.03	0.78
Ile	17.8	10.6	0.76	0.05	0.04	0.28	0.00
Leu	36.0	22.6	0.67	0.10	0.08	0.35	0.00
Lys	37.5	24.5	0.62	0.76	0.92	−0.28	0.18
Met	5.34	4.33	0.30	0.04	1.08	−4.63	0.00
Phe	20.0	12.9	0.63	0.03	0.03	0.03	0.56
Pro	–	–	–	0.92	2.76	−1.58	0.00
Ser	25.9	19.0	0.44	0.25	0.52	−1.04	0.00
Thr	28.2	19.3	0.55	0.24	0.27	−0.14	0.42
Tyr	17.3	8.89	0.96	0.06	0.05	0.18	0.02
Val	26.4	15.7	0.75	0.25	0.14	0.83	0.00
Total amino acids [mg/g Cell dry weight (CDW)]	448	306	0.55	43.2	52.1	−0.27	0.03
Total protein content [mg/gCDW]	540	390	0.47	–	–	–	–

While methanol utilization enzymes were up-regulated at the transcriptional level and thus increased at the protein level, the second major class of more abundant proteins (ribosomal proteins) were not transcriptionally regulated. This finding indicates an efficient post-transcriptional regulation mechanism, as described for *S. cerevisiae* [57], and a higher steady state demand of translational capacity on methanol. This last observation is supported also by a higher total protein content of biomass grown on methanol/glycerol and by the up-regulation of amino acid synthesis pathways. It remains to be elucidated whether this higher translational capacity on methanol is related to the observed higher recombinant protein production capacity of methanol-based expression strains. The general upregulation of protein synthesis in methanol-induced cultures did not coincide with a higher abundance of enzymes in the protein folding machinery. An accumulation of misfolded proteins as a result of heterologous gene expression has been observed many times in recombinant *P. pastoris*, leading to UPR activation (reviewed by Puxbaum et al*.* [58]) but appears to be absent in non-recombinant *P. pastoris* cultivated on methanol.

### Protein folding, secretion, and degradation pathways are not affected by methanol as substrate

While we and others have observed a transient up-regulation of the UPR immediately following methanol induction [59, 60], no such regulation pattern was noticed in the methanol-adapted cells in steady state in the present study, thus ruling out the possibility that a permanently induced UPR positively influences recombinant protein production in methanol-grown cells. Contrary to Liang et al*.* [16], who detected up-regulation of endoplasmic reticulum protein processing, N-glycan biosynthesis, and protein export pathways when comparing recombinant protein secreting *P. pastoris* in chemostats with methanol as substrate, we did not see any changes in protein folding, secretory pathway, N-glycosylation, or proteasome both at the proteome and transcriptome level in the non-expressing strains in our study (Additional file 1). Protein synthesis, however, was obviously up-regulated on methanol, as described above.

### High levels of methanol utilization enzymes require overproduction of vitamins and cofactors

#### Alcohol oxidase requires high riboflavin synthesis

AOX, which catalyzes the first reaction of methanol utilization, is a homooctamer with flavin adenine dinucleotide (FAD) as non-covalently bound prosthetic group. When methylotrophic yeast cells grow on methanol, AOX can account for up to 30 % of total cellular protein [61], and the FAD content of AOX alone amounts to 1.7 mg/g biomass. AOX predominantly oligomerizes in the peroxisomal matrix of methylotrophic yeasts [62, 63]. Experiments with *H. polymorpha* and *P. pastoris* revealed that insertion of FAD is an essential step prior to the assembly of AOX [63].

**Fig. 7:**
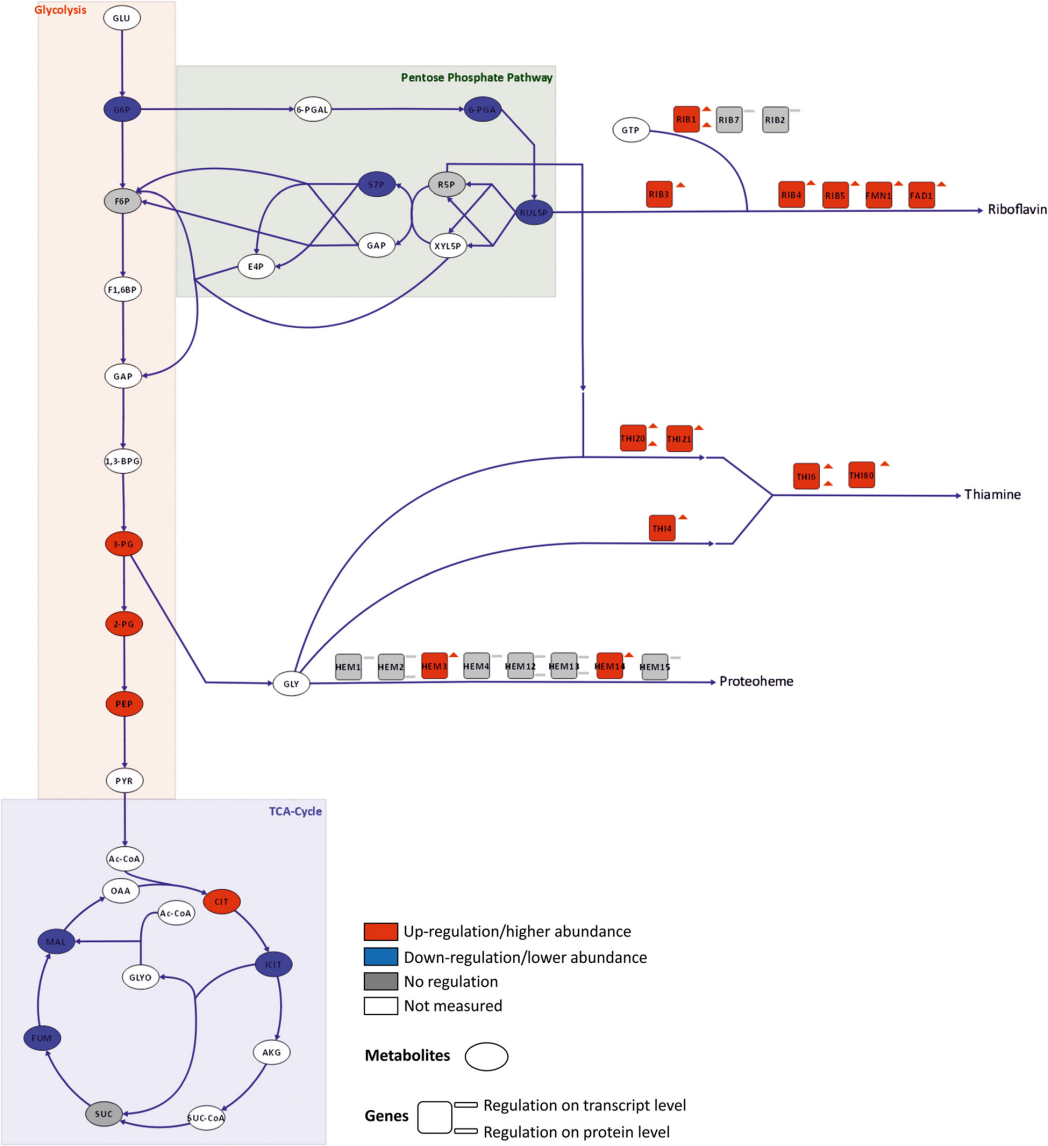
Differential regulation of riboflavin, thiamine, and heme synthesis pathways comparing methanol/glycerol- and glucose-grown cells. Visualization of changes in transcript, protein, and metabolite levels. For an explanation of the symbols see legend to Fig. 4

Almost the entire pathway leading to FAD is transcriptionally up-regulated when methanol is present (Fig. 7 and Additional files 1 and 2): *RIB1* and *RIB3* encode the first steps of the riboflavin biosynthesis pathway (with GTP and ribulose-5-phosphate as precursors, respectively), while *RIB4* and *RIB5* code for the last enzymes in the pathway. The induction of the riboflavin pathway during growth on methanol has been previously observed [16], but was not linked to AOX biosynthesis. Via the up-regulated *FMN1*, riboflavin is converted to flavin mononucleotide (FMN), a strong oxidizing cofactor of mitochondrial NADH-dehydrogenases (which, however, are not regulated). The generation of FAD from FMN is catalyzed by Fad1, which is strongly transcriptionally up-regulated (~8-fold). The bulk of FAD apparently goes into AOX as other cellular flavoproteins [64] are rather unaffected during growth on methanol with the FAD-requiring Gut2, Hem14 (both up-regulated), Pox1, Fmo1-1, and Fre2 (all three down-regulated) as exceptions. We observed no changes in free riboflavin, indicating that flux to riboflavin is up-regulated upon its high demand while synthesis is tightly regulated by its free intracellular concentration as described by Marx et al*.* [65].

### Thiamine synthesis is strongly up-regulated due to demand of peroxisomal transketolases

There is also significant up-regulation of genes involved in thiamine (vitamin B1) and thiamine pyrophosphate (TPP) biosynthesis (*THI20*, *THI6*, *THI80*, *THI4*, *THI13*, *THI73*, *THI21*; Fig. 7 and Additional files 1 and 2) in cells grown on methanol. We have shown before that severe thiamine limitation is required for the induction of *THI13* [66], indicating that induction of the methanol utilization pathway leads to intracellular thiamine deficiency.

TPP, the active derivative of thiamine, is the co-factor of decarboxylases, transketolases, and phosphoketolases. The homodimeric enzymes bind one Mg^2+^ ion and one TPP per subunit. In *P. pastoris* cultures grown on methanol, upregulation of TPP biosynthesis coincided with the high abundance of the TPP-containing enzymes Das1 and Das2, catalyzing the fixation of formaldehyde to XYL5P in the peroxisome. In this study, Das1 and Das2 had very high changes in transcript levels (~68-fold and 46-fold up-regulation, respectively) and one of the highest changes in protein level (~6.5-fold and 4.7-fold, respectively). On the contrary, the level of cytosolic transketolase (Tkl1) was unaffected. Thus, we conclude that the strong induction of DAS1/2 led to a limitation of thiamine availability which was compensated by induction of the thiamine synthesis pathway.

### High nicotinamide levels are required for formaldehyde detoxification

In the methanol dissimilation pathway, formaldehyde is oxidized to carbon dioxide by two consecutive reactions catalyzed by formaldehyde dehydrogenase and formate dehydrogenase. On methanol, both enzymes are strongly increased both on transcript (5-fold and 19-fold, respectively) and protein (formaldehyde dehydrogenase not quantified, formate dehydrogenase 5-fold) levels. The two enzymes, which are mainly located to the cytosol, are required for detoxification of formaldehyde and formate and both use nicotinamide adenine dinucleotide as cofactor. The generated NADH provides energy for growth on methanol. In this respect, the total amount of nicotinamide in cells grown in the presence of methanol was nearly 10-fold higher than in the glucose-grown cells, and total NAD content is approx. 50 % higher. Expression of *NMA1*, encoding nicotinic acid mononucleotide adenylyltransferase, which is involved in the *de novo* biosynthesis of NAD as well as in the NAD salvage pathway [67], was up-regulated 2.4-fold.

### Heme synthesis is up-regulated upon catalase demand for peroxide detoxification

Toxic H_2_O_2_ and formaldehyde are generated in the first step of methanol metabolism. The peroxisomal enzyme catalase, which is involved in the detoxification of H_2_O_2_, is transcriptionally up-regulated when methanol is present. Properly folded catalase incorporates a heme cofactor with an iron ion in the center, and needs to tetramerize to become active [63]. *CTA1* expression is up-regulated on methanol and, consequently, expression of almost all heme biosynthesis genes was up-regulated, including the rate-limiting steps *HEM2* and *HEM3* (log_2_FC +0.43 and +0.54). Pet18, a heme oxygenase-like protein, was also up-regulated at both the transcript and protein levels. Heme oxygenases catalyze the degradation of heme and produce iron. Down-regulation (0.6-fold) of a low-affinity Fe(II) transporter (*FET4-2*) and up-regulation (1.65-fold) of *FTH1-1*, a putative high affinity iron transporter involved in intravacuolar iron storage, points towards low iron levels in the presence of methanol.

### On methanol, the general lipid metabolism is altered to allow peroxisome formation at the expense of lipid droplets

Environmental conditions and nutritional modifications often have dramatic effects on the composition of cellular membranes, which becomes apparent in lipid composition and regulation of lipid metabolism. Major key enzymes of lipid-related pathways were apparently not affected when culture conditions varied between the supply of glucose or methanol/glycerol. Important components of biological membranes, sterols and phospholipids, were elevated only slightly in methanol/glycerol-grown cells (Table 5). The observed increase of building blocks for membranes can most likely be explained by the enhanced occurrence of internal membranes. The total amount of peroxisomes was strongly increased in methanol/glycerol-grown cells which caused a weak effect on the total amounts of phospholipids and sterols of internal membranes. Wriessnegger et al*.* [44, 68] already showed in previous work that utilization of glucose or methanol as the sole carbon source does not lead to major differences in the distribution of phospholipids, although the culture conditions and sampling points were not the same as in the present study. The slight increase in the total amount of phospholipids observed here was not matched by any significant regulation of lipid biosynthetic genes involved in the complex pathways of phospholipid formation, except for *INO1* and *OPI3*, which were both down-regulated (log_2_FC of −1.38 and −0.89). The pattern of fatty acids from methanol-grown cells as well mostly resembled glucose-grown cells, although some minor changes were detected. A decrease in oleic acid (C18:1) by roughly 20 % was accompanied by an increase in palmitic acid (C16:0), palmitoleic acid (C16:1), and linolenic acid (C18:3). Again, the influence of the intracellularly predominant peroxisomal membranes most likely was the reason for the observed changes of bulk membrane fatty acid composition.

**Table 5:** Glycerophospholipid, non-polar lipid (TG, triacylglycerol; SE, steryl esters), unesterified ergosterol, and free and total fatty acid content in total cell extracts of *Pichia pastoris* grown on glucose (GAP) or methanol (AOX) as the sole carbon source. Data are listed as μg lipid/mg Wet Cell Weight which have been calculated from at least two independent experiments with standard deviation (±). Significance was estimated by Student’s *t*-test (two tailed, unpaired)

	Glucose	Methanol/glycerol	*P* value
Glycerophospholipids	8.07 ± 0.14	8.92 ± 0.30	0.01
Non-polar lipids			
TG	3.16 ± 0.71	1.47 ± 0.28	0.06
SE	0.22 ± 0.03	0.30 ± 0.03	0.07
Free ergosterol	1.67 ± 0.06	1.83 ± 0.18	0.20
Free fatty acids	2.11 ± 0.49	3.38 ± 0.72	0.09
Total fatty acids			
C16:0	1.22 ± 0.03	1.43 ± 0.16	0.01
C16:1	0.75 ± 0.01	0.85 ± 0.06	0.003
C18:0	0.33 ± 0.02	0.34 ± 0.09	0.67
C18:1	4.28 ± 0.18	3.30 ± 0.31	0.0002
C18:2	3.26 ± 0.14	3.45 ± 0.30	0.21
C18:3	0.96 ± 0.04	1.30 ± 0.11	0.0001
∑ of fatty acids	10.79 ± 0.40	10.67 ± 1.01	0.79

**Fig. 8:**
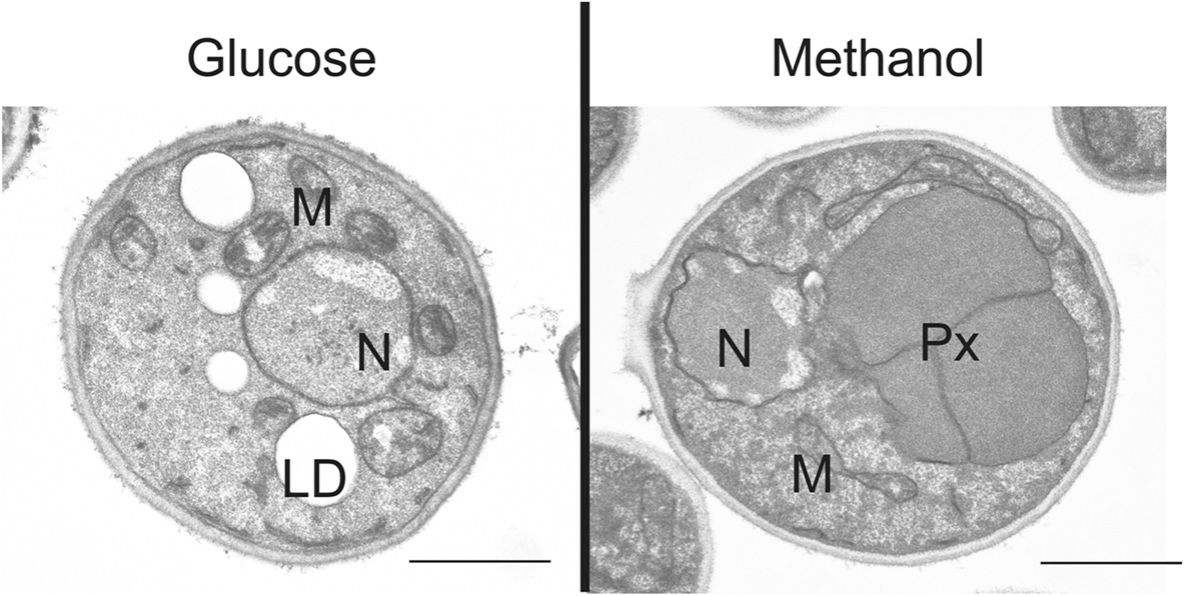
Electron microscopy of *P. pastoris* grown on glucose or methanol. Cells were cultivated in complex media containing either glucose or methanol as the sole carbon source until they reached the late exponential growth phase. N, Nucleus; M, Mitochondria; LD, Lipid droplet; Px, Peroxisome. Scale bar: 1 μm

The strongest effect on lipid classes resulting from cultivation on different carbon sources was on triacylglycerols (TAG), the major non-polar lipid of *P. pastoris*. Both TAG synthases, *DGA1* and *LRO1*, were transcriptionally down-regulated on methanol/glycerol (log_2_FC −1.07 and −0.43). As a direct result, TAG were reduced in methanol/glycerol-grown cells by more than 50 % (Table 5). The significant decrease of TAG was accompanied by a severe reduction of lipid droplets in *P. pastoris* cultivated on methanol, which was observed by electron microscopy (Fig. 8). While the amount of TAG was severely reduced, precursors of TAG (diacylglycerols and free fatty acids) were increased by approximately 40 %. Upon mobilization of TAG by TAG lipases, activated fatty acids could serve as substrates either for β-oxidation or as building blocks for membrane formation. In comparison to glucose we observed on methanol/glycerol a down-regulation of transcripts encoding β-oxidation relevant genes as well as TAG forming enzymes, which was not followed at the protein level. Notably, it has been previously shown that genes involved in fatty acid utilization are differentially regulated upon using glycerol or glucose as the carbon source, and depend on substrate availability [37] (Additional file 2). The utilization of methanol enables *P. pastoris* cells for proper growth based on energy supply by alcohol oxidation, but apparently does not provide excess carbons to be incorporated in storage material. Therefore, non-polar lipid synthesizing enzymes are down-regulated. As a direct consequence, no alternative supply of fatty acids may be available and β-oxidation relevant enzymes are shut down as well because of the limited substrate available.

*ERG20*, encoding farnesyl pyrophosphate synthetase, is the only lipid biosynthetic gene which was found to be up-regulated when comparing glucose to methanol-grown cells. Erg20 is part of the sterol biosynthetic pathway, which is composed of more than 20 enzymes. However, all other sterol biosynthetic genes remained transcriptionally unaffected. Erg20 is located at an important branching point of this biosynthetic pathway. The product of the Erg20 catalyzed reaction, farnesyl pyrophosphate, cannot only serve as a substrate for the formation of structural lipid compounds which is one of the major routes, but can be directed towards several other pathways, among them heme biosynthesis. As the formation of heme was found to be transcriptionally up-regulated to serve as a prosthetic group of catalase, we anticipated that *ERG20* was up-regulated predominantly to provide sufficient substrate for the *de-novo* formation of heme.

## Conclusions

Methylotrophy is a unique ability of microorganisms to live on C1 molecules that requires efficient pathways to form C-C bonds and to oxidize C1 compounds via toxic intermediates. This systems level investigation provides comprehensive insight into regulatory and metabolic specificities of the methylotrophic yeast *P. pastoris*. Co-regulation of enzymes with AOX and DAS at the transcript and protein level allowed us to identify in detail the putative pathway for XYL5P regeneration during methanol assimilation. We revealed that the xylulose-monophosphate cycle is employing a specialized set of methanol-induced enzymes located in the peroxisome, rather than the PPP proteins, which are essentially not transcriptionally or translationally regulated in this study. For this purpose, *P. pastoris* has acquired a second copy of the relevant genes, each adjacent to the canonical PPP gene. During growth on methanol, the peroxisomes also harbor an active glyoxylate cycle, while the TCA cycle flux is reduced, indicating that methanol dissimilation is a major source for NADH and energy production on this substrate. Furthermore, growth on methanol/glycerol leads to a higher amino acid synthesis rate and a higher translational capacity which is reflected by a higher total protein content and may indicate a higher capacity for production of heterologous proteins as well. The observed changes in lipid metabolism can be explained by the high abundance of peroxisomes and the absence of lipid droplets in methanol-grown *P. pastoris*. The methylotrophic lifestyle reflects a low energy status, thus impeding lipid storage. During growth on methanol, the methanol utilization enzymes are produced in high amounts. Consequently, the biosynthetic pathways for the corresponding prosthetic groups and co-enzymes are also strongly up-regulated. Up-regulation of the pathways to riboflavin, thiamine, nicotinamide, and heme clearly indicates their high steady state demand in methanol-grown cells.

This work provides a unique data set on the methylotrophic metabolism of *P. pastoris*, and enables the redefinition of the methanol assimilation pathway. These findings will also have major impact on the understanding and evolution of methylotrophy in other yeasts.

## Methods

### Strains & chemostat cultivation

The chemostat cultivations were performed in a 1.4-L bioreactor (DASGIP Parallel Bioreactor System, Germany) with a working volume of 400 mL.

Briefly, 100 mL pre-culture medium (per liter: 10 g yeast extract, 20 g peptone, 10 g glycerol) were inoculated with 750 μL cryostock of *P. pastoris* CBS7435 and grown at 28 °C and 150 rpm overnight. This culture was used for inoculation of the bioreactor at an optical density (OD_600_) of 1.0. After a batch phase of approximately 24 h, the cells were grown in carbon-limited chemostats with a dilution rate of 0.1 h^−1^ for at least seven residence times before taking the samples. For each condition, three independent chemostat cultivations were performed. Temperature, pH, and dissolved oxygen were maintained at 25 °C, 5.0 (with 8 M KOH) and 20 % (by controlling the stirrer speed and inlet air), respectively.

Batch medium contained per liter: 39.9 g glycerol, 1.8 g citric acid, 12.6 g (NH_4_)_2_HPO_4_, 0.022 g CaCl_2_·2H_2_O, 0.9 g KCl, 0.5 g MgSO_4_·7H_2_O, 2 mL biotin (0.2 g L^−1^), 4.6 mL trace salts stock solution. The pH was set to 5.0 with 32 % (w/w) HCl.

Chemostat medium (Glucose) contained per liter: 55 g glucose·H_2_O, 2.3 g citric acid, 21.75 g (NH_4_)_2_HPO_4_, 0.04 g CaCl_2_·2H_2_O, 2.5 g KCl, 1.0 g MgSO_4_·7H_2_O, 2 g biotin (0.2 g L^−1^), and 2.43 g trace salts stock solution. The pH was set to 5.0 with 32 % (w/w) HCl.

Chemostat medium (methanol/glycerol) contained per liter: 57 g glycerol (86 %), 8.5 g methanol (100 %), 2.3 g citric acid, 21.75 g (NH_4_)_2_HPO_4_, 0.04 g CaCl_2_·2H_2_O, 2.5 g KCl, 1.0 g MgSO_4_·7H_2_O, 2 g biotin (0.2 g L^−1^), and 2.43 g trace salts stock solution. The pH was set to 5.0 with 32 % (w/w) HCl.

Trace salts stock solution contained per liter: 6.0 g CuSO_4_·5H_2_O, 0.08 g NaI, 3.0 g MnSO_4_·H_2_O, 0.2 g Na_2_MoO_4_·2H_2_O, 0.02 g H_3_BO_3_, 0.5 g CoCl_2_, 20.0 g ZnCl_2_, 5.0 g FeSO_4_·7H_2_O, and 5.0 mL H_2_SO_4_ (95–98 % w/w).

### Sampling and quenching

For transcriptomics, 9 mL of culture were added to 4.5 mL of freshly prepared pre-chilled (−20 °C) fixing solution (5 % v/v phenol in ethanol abs.), mixed, and 1.5 mL were aliquoted into ribolyzer tubes and centrifuged at 13,000 rpm for 1 min at 4 °C. The supernatant was discarded and the tubes containing the fixed cell pellets were immediately stored at −80 °C. For protein analysis, 2 mL of culture were centrifuged and the cell pellet was stored at −80 °C. The supernatant was also stored at −80 °C for analysis of extracellular metabolites.

Samples for analysis of intracellular metabolites were taken immediately by using a pump. Approximately 50 mL fermentation broth were quenched in 200 mL of 60 % (v/v) methanol at −27 °C. After quenching, 2 mL of quenched cells (corresponding to approximately 10 mg biomass per filter) were filtered with cellulose acetate filter (0.45 μm, Satorius Biolab Products) using a vacuum pump.

The cells were washed once with cold 60 % (v/v) methanol and then the filter was kept on dry ice. Using two filtration units (Polycarbonat Filter Holders, Satorius Lab Technologies Product), 6 samples per chemostat cultivation were taken.

Biomass was determined by drying duplicates of 2 mL chemostat culture to constant weight at 105 °C in pre-weight beakers.

### Total protein determination

Cell pellets from 2 mL chemostat culture were washed with 0.9 % NaCl and resuspended in 1 mL of PBS (pH 7.0). The protein extraction was done accordingly to Verduyn et al*.* [69], by addition of NaOH and incubation at 95 °C. After incubation, 0.8 M HCl were added and cell debris were collected via centrifugation. The supernatant was used for the determination of the total protein content using Bradford. The total protein content was related to the yeast dry mass (%).

### Lipid analysis

Lipid extraction from *P. pastoris* chemostat samples was performed as described by Folch et al*.* [70]. For quantitative determination of non-polar lipids (TG, SE), free fatty acids and free ergosterol, lipid extracts were loaded to Silica Gel 60 plates (Macherey-Nagel, Düren, Germany) and chromatograms were developed in an ascending manner by using the solvent system light petroleum/diethyl ether/acetic acid (70:30:2; per vol.) for approximately the first third of the distance. Subsequently, plates were briefly dried and further developed using the solvent system light petroleum/diethyl ether (49:1; per vol.) until the solvent front reached the top of the plate. Unesterified sterols and steryl esters were quantified densitrometrically using a TLC scanner (Camag TLC Scanner 3) at 275 nm using ergosterol as standard. Other lipids were irreversibly stained by dipping the TLC plates into a charring solution (0.63 g MnCl_2_·4H_2_O, 60 mL water, 60 mL methanol, and 4 mL concentrated sulfuric acid) and heated at 100 °C for 30 min. Densitrometric scanning was performed at a wavelength of 400 nm, and lipids were quantified with ergosterol, oleic acid, or triolein as standard.

For estimation of total amounts of glycerophospholipids separate bands from non-polar lipid analysis (see above) were used. Glycerophospholipids were visualized on plates by reversible staining with iodine vapor, scraped off, and subjected to quantification by the method of Broekhyuse [71].

Analysis of total fatty acids was achieved by conversion to methyl esters by methanolysis using 2.5 % sulfuric acid in methanol and heating at 85 °C for 90 min. FAMEs (fatty acid methyl ester) were extracted twice in a mixture of light petroleum and water (3:1; v/v.) and subjected to gas liquid chromatography (Hewlett-Packard 6890 Gas-chromatograph) using an HP-INNOWax capillary column (15 m × 0.25 mm i.d. × 0.50 μm film thickness) with helium as carrier gas. Fatty acids were identified by comparison to commercially available fatty acid methyl ester standard mix GLC-68B (NuCheck, Inc., Elysian, MN, USA) and quantified by using pentadecanoic acid (Sigma) as an internal standard.

### Microarrays and data analysis

The RNA was isolated from chemostat sample cells using TRI reagent according to the supplier’s instructions (Ambion, USA). RNA integrity was analyzed using RNA nano chips (Agilent). In-house designed *P. pastoris*-specific oligonucleotide arrays (AMAD-ID: 034821, 8x15K custom arrays, Agilent) were used [20, 32]. cRNA synthesis, hybridization, and scanning were performed according to the Agilent protocol for two-color expression arrays. Each sample was hybridized against an RNA reference pool sample in dye swap. The microarray data was not background normalized. Normalization steps and statistical analysis of microarray data included removal of color bias using locally weighted MA-scatterplot smoothing (LOESS) followed by between array normalization using the “Aquantile” method. The *P* values associated with the differential expression values were calculated using a linear model fit (limma R package), subsequently they were adjusted for multiple testing using the method of Benjamini and Yekutieli [72] using the BY method of limma R package. To identify differentially expressed genes, the following criteria were applied: fold change cut-off of at least 1.5 > FC >1/1.5 and adjusted *P* value <0.05. All steps were performed using the R software package [73], and the limma package. Transcriptomics data were deposited at Gene Expression Omnibus with the accession number GSE67690. Data can be accessed with following link http://www.ncbi.nlm.nih.gov/geo/query/acc.cgi?token=stopswyszunfkf&acc=GSE67690.

### Proteomics

#### Cell lysis and sample preparation

Cells were lysed with glass beads as described by Dragosits et al. [24] in 100 mM triethylammoniumbicarbonate (TEAB) buffer, containing 30 mM tris(2-carboxyethyl)phosphine hydrochloride and 2 % SDS. After incubation for 45 min at 56 °C (to reduce cysteine bridges) cellular proteins were extracted with chloroform/methanol, dried, dissolved in TEAB buffer, and digested with trypsin. Tandem Mass Tag (Thermo Scientific) labelling was performed as described by Pichler et al. [74] following the manufacturer’s protocol.

#### 2D-LC and MS analysis

Samples were separated by high pH C18 HPLC applying an elution gradient of 12.5–80 % acetonitrile at pH 10 (200 mM ammonium formiate). Eighteen fractions were collected, partially pooled and applied to a C18 nano-column on a Bruker maxis 4G ETD QTOF LC-MS instrument, and separated with a 5–32 % acetonitrile gradient with 0.1 % formic acid (followed by a 32–80 % gradient to elute large peptides). The mass spectrometer was equipped with the captive spray source (1350 V capillary voltage, 3 L/min dry gas). Mass spectrometry scans were recorded in DDA mode (range: 150–2200 Da) and the 10 highest peaks were selected for fragmentation. The mass spectrometry proteomics data have been deposited to the ProteomeXchange Consortium via the PRIDE partner repository with the dataset identifier PXD002036.

### Peptide/protein identification

The software Mascot was used for the identification of peptides and proteins by matching the observed spectra with a database containing unique *P. pastoris* protein sequences. Mascot uses the MOWSE (MOlecular Weight SEarch) score: the more matches, the higher the peptide score. Protein scores are the sum of the peptide scores. Protein identification requires the match of at least two independent peptides with a score of >25.

### Data processing

For quantitative analysis of the proteomics data, the software Isobar Version 1.7.5 was used [75]. Mascot identification and quantification data were normalized using Isobar's default normalization method, which corrects for differences in reporter channel median intensities. Intensity measurement noise was corrected with a noise model comparing identical samples in multiple channels. For obtaining the protein ratios, Isobar calculates a weighted average of the peptide spectra after eliminating outliers. Comparing different distributions showed that a t-distribution fitted the random protein ratio distribution of our data best, and was selected for *P* value calculation of differentially expressed proteins.

Three biological replicates, with two technical replicates each, had been analyzed leading to six replicate data sets of both growth conditions. For every identified peptide, Isobar calculated the log_2_ of the ratio between the methanol/glycerol samples and the glucose samples (log_2_ FC) and the *P* value. Peptides with ion intensity values smaller then 300 and protein ratios deriving from single peptide spectra were excluded from the analysis, as well as proteins that were identified only in one or two replicates; 1,066 proteins fulfilled those criteria.

Proteins meeting the following criteria were defined as significantly changed between growth conditions: |mean FC| >1.5 and *P* values <0.1 or 1.3 < |mean FC| ≤1.5 and *P* value <0.05. Proteins with |mean FC| ≤1.3 and *P* value >0.05 were defined as not changed between the two growth conditions. To further increase stringency of evaluation we defined that >50 % of the replicates in which a given protein could be identified must have the same regulatory characteristics. Proteins that did not fulfill these criteria were not further considered. From the 1,066 proteins identified, 575 could be quantitatively evaluated.

### Metabolomics

#### Extraction and measurement of intracellular metabolites

For the measurement of intracellular concentrations of free metabolites quenched cells on cellulose acetate filters were used. Prior to the extraction, uniformly labelled ^13^C internal standard was added to the samples. Free intracellular metabolites were extracted by addition of 4 mL boiling HPLC grade ethanol (82 %; v/v; tempered at 85 °C). After addition of the boiling ethanol the quenched cells were immediately suspended by vortexing for 30 s. Suspended cells were heated for 3 min in total at 85 °C using a water bath. After 1.5 min of extraction samples were vortexed for 10 s and put back to the water bath at 85 °C. After 3 min of extractions extracted cells were immediately cooled down on dry ice. The cooled sample was then centrifuged to remove cell debris (10 min, −20 °C, 4000 *g*). The ethanolic extract was decanted into a fresh cooled 15 mL tube and kept on dry ice until sample preparation for LC-MS/MS and GC-MS/MS analysis. LC-MS/MS analysis of free intracellular metabolites was performed according to Klavins et al. [76], whereas GC-MS/MS analysis of sugar phosphates was performed after automated derivatization via ethoximation followed by trimethylsilylation. Both methods employed quantification by external calibration utilizing a uniformly ^13^C-labeled ethanolic extract of *P. pastoris* for internal standardization [77].

#### Detection of sedoheptulose-1,7-bisphosphate in cell extracts of *P. pastoris*

Acetonitrile, water, and formic acid (all LC-MS grade) were purchased at Sigma-Aldrich. *P. pastoris* cells were grown in glucose- or methanol-limited conditions in bioreactors. A set of three samples from glucose-fed cellular extracts was compared to a set of three methanol-grown samples. Each sample was derived from a separate biological replicate. After extraction (see above) the samples were stored at −80 °C until analysis. 500 μL of the sample were evaporated to dryness using a Savant SPD 121P SpeedVac Concentrator (Thermo Scientific). The residues were reconstituted in 100 μL water and directly analyzed.

Liquid chromatography separation was performed on a Hypercarb 150 × 2.1 mm, 3 μm particle size column (Thermo Scientific) with a Hypercarb guard cartridge (10 × 2.1 mm, 3 μm) using a 1260 BinPumpSL (Agilent Technologies) combined with a CTC Pal autosampler (CTC Analytics AG). The flow rate was 250 μL min^−1^ and the column oven was set to 40 °C. Sample injection volume was 5 μL. Mobile phase A was 100 % water, whereas mobile phase B contained 80 % acetonitrile, 10 % water, and 10 % formic acid. A gradient was applied as follows: starting conditions of 1 % B were held for 2.5 min and then increased to 40 % within 14 min. This composition was held for 1 min, before returning to 1 % B in 0.1 min for re-equilibration. The total analysis time was 20 min.

An Agilent 6220 LC-TOFMS system equipped with a dual-ESI-Source was used for the LC-MS analysis. Source parameters for negative mode were set as follows: 350 °C gas temperature, 10 L min^-1^ drying gas flow, 25 psig nebulizer gas pressure, 3500 V capillary voltage, 140 V fragmentor voltage, and 60 V skimmer voltage. The mass spectrometer was operated in the 2 GHz mode (extended dynamic range) recording the mass range from 50 to 1000 m/z with an acquisition rate of 1.03 spectra s-1 (9644 transients per spectrum). Data evaluation was performed using the Agilent MassHunter Qualitative Analysis B.07.00.

The identification of sedoheptulose-1,7-bisphosphate was confirmed by comparing the signals obtained in the samples to a standard which was provided by Amy A. Caudy (University of Toronto, Canada). The difference between the measured accurate mass and the calculated exact mass was below 2 ppm for all samples where sedoheptulose-1,7-bisphosphate was detected.

### ^13^C-Metabolic flux analysis

^13^C-labelling experiments were performed as described in Baumann et al. [21]. The cells grew in a chemostat at a constant growth rate of 0.1 h^−1^ on a mixture of 20 % fully ^13^C-labelled substrate and 80 % naturally labelled substrate, either glucose or methanol/glycerol. The labelling pattern of protein-bound amino acids was determined via GC-MS. The GC-MS spectra were used for the calculation of mass distribution vectors of the protein bound amino acids [78]. The Matlab-based software package Openflux was used for ^13^C-Metabolic flux analysis. For the calculation standard settings were applied [79]. The flux calculation was performed with a stoichiometric model of *P. pastoris* central carbon metabolism. The model is analogous to the model already published by Jorda et al*.* [28]. As a constraint, the labelling pattern of protein-bound amino acids and the calculated uptake and segregation rates of extracellular metabolites were used.

### Isolation and proteome characterization of the peroxisomes

*P. pastoris* cells were cultivated on glucose (YPD) or methanol (YPM) until they reached the late logarithmic growth phase. Cellular fractions enriched of highly pure peroxisomes were isolated following the procedure which had previously been established for *P. pastoris* by Wriessnegger et al*.* [44]. Isolated peroxisomes from methanol- and glucose-grown cells and the respective homogenates were evaluated for specific marker protein enrichment by Western blots (Additional file 3) and subjected to proteomics identification.

Samples were analyzed with a nano LC system as described above in 2D-LC and MS analysis. A standard 180 min gradient, using 0.1 % formic acid and 80 % acetonitrile as solvents, was applied. Data interpretation was performed manually (quantification) using DataAnalysis 4.0 and the files were converted to XML files for protein identification. XML files are suitable for performing a MS/MS ion search with ProteinScape (Bruker software, MASCOT embedded). At least two peptides and a MASCOT score of 30 were minimum thresholds for a positive hit. For quantification, the extracted ion chromatograms of the most intense peptides of each protein were integrated and peak areas were calculated. The sum of peak areas of each protein quantified of peroxisomal preparations was set in relation to the sum of peak areas of homogenate samples. Thus, a value higher than 1 reflects a relative enrichment in comparison to Aox1 in the peroxisomal preparation and a value lower than 1 shows a lower abundance in the peroxisomal fraction in comparison to Aox1. Peptide and protein hits, and the peak areas of peptides used for quantification are provided in Additional file 4. The mass spectrometry proteomics data were deposited in the ProteomeXchange Consortium via the PRIDE partner repository with the dataset identifier PXD002831.

### Electron microscopy

Cells were cultivated at 25 °C with shaking at 150 rpm in baffled flasks using YPD until reaching the late exponential phase. Washed cells were fixed for 5 min in a 1 % aqueous solution of KMnO_4_ at room temperature, washed with double distilled water, and fixed again in a 1 % aqueous solution of KMnO_4_ for 20 min. Fixed cells were washed four times in distilled water and incubated in 0.5 % aqueous uranyl acetate overnight at 4 °C. Samples were then dehydrated for 20 min, in a graded series of 50 %, 70 %, 90 %, and 100 % ethanol, each. Pure ethanol was then changed to propylene oxide and specimens were gradually infiltrated with increasing concentrations (30 %, 50 %, 70 %s and 100 %) of Agar 100 epoxy resin mixed with propylene oxide for a minimum of 3 h per step. Samples were embedded in pure, fresh Agar 100 epoxy resin and polymerized at 60 °C for 48 h. Ultrathin 80-nm sections were stained for 3 min with lead citrate and viewed with a Philips CM 10 transmission electron microscope.

## Additional files

Additional file 1:
**Transcriptomic, proteomic, and metabolomic regulation of** ***P. pastoris*** **during methylotrophic growth.** Containing the following eight sheets: Summary Omics Data: number of significantly regulated genes, proteins or metabolites (e.g. “up” refers to up-regulation in methanol/glycerol compared to glucose). Transcriptomics and proteomics: Average fold changes and *P* values of transcriptomics and proteomics comparing *P. pastoris* cultivated with methanol/glycerol or glucose as carbon source in chemostat. Average values derive from three biological replicates per condition. Metabolomics: Average fold changes and *P* values of metabolomics measurements comparing *P. pastoris* cultivated with methanol/glycerol or glucose as carbon source in chemostat cultivations. Average values derive from three biological replicates per condition. Co-regulation (related to Fig. 1 in the text): Regulation of the 575 gene-protein pairs with transcriptomics and proteomics data available and assignment to regulatory groups. Central carbon metabolism (related to Fig. 4 in the text): Average fold changes and *P* values of transcriptomics, proteomics, and metabolomics measurement depicted in Fig. 4. Amino acid metabolism (related to Fig. 6 in the text): Average fold changes and *P* values of transcriptomics, proteomics, and metabolomics measurement depicted in Fig. 6. Vitamin biosynthesis (related to Fig. 7 in the text): Average fold changes and *P* values of transcriptomics, proteomics, and metabolomics measurement depicted in Fig. 7. Peroxisomal gene regulation: Average fold changes and *P* values of transcriptomics and proteomics for all mentioned peroxisomal genes. Average values derive from three biological replicates per condition. (XLSX 2348 kb) Additional file 2:
**Comparison of gene regulation in** ***P. pastoris*** **cultivated with methanol/glycerol or glucose as carbon source in chemostat to transcriptomics data obtained in fed batch cultivation with methanol glycerol or glucose as carbon source.** Average fold changes and *P* values of regulation patterns for methanol vs glycerol and methanol vs glucose 1 h our after starting the methanol feed are shown. Average values derive from three biological replicates per condition. Containing the following four sheets: Description: Experimental setup of the fed batch cultivations, description, and discussion of observed similarities and differences in gene regulations. Upregulated_methanol: Comparison of all genes upregulated in *P. pastoris* cultivated with methanol/glycerol or glucose as carbon source in chemostat to transcriptomics data obtained by analyzing methanol vs glycerol and methanol vs glucose fed batches. Downregulated_methanol: Comparison of all genes downregulated in *P. pastoris* cultivated with methanol/glycerol or glucose as carbon source in chemostat to transcriptomics data obtained by analyzing methanol vs glycerol and methanol vs glucose fed batches. All_data: Transcriptomics data of Additional file 1 (*P. pastoris* cultivated with methanol/glycerol or glucose as carbon source in chemostat) compared to transcriptomics data obtained by analyzing methanol vs glycerol and methanol vs glucose fed batches. (XLSX 1178 kb) Additional file 3:
**Enrichment of the peroxisomal marker protein Pex3p in the peroxisomal fraction.** (PDF 271 kb) Additional file 4:
**Proteomic identification and quantification of methanol metabolic enzymes and control proteins in peroxisomal fractions and homogenates of** ***P. pastoris*** **cells grown on methanol.** Containing the following three sheets: Protein hits: contains all identified proteins that met the threshold in at least one sample, with their respective MASCOT scores, number of peptides, and percent sequence coverage. Peptide hits: list of all identified peptides, their MASCOT scores, mass and charge values, and intensities. Peptides used for quant + areas: lists all peptides of the proteins in Table 3 that were used for quantification, and their respective peak areas in the different samples. (XLSX 879 kb)

## Abbreviations

AOX: Alcohol oxidase
CDW: Cell dry weight
DAK: Dihydroxyacetone kinase
DAS: Dihydroxyacetone synthase
DHAP: Dihydroxyacetone phosphate
FAD: Flavin adenine dinucleotide
Fba1-2: Fructose-1,6-bisphosphate aldolase
FMN: Flavin mononucleotide
GAP: Glyceraldehyde-3-phosphate
H_2_O_2_: Hydrogen peroxide
PPP: Pentose phosphate pathway
PTS: Peroxisomal targeting signal sequence
Rki1-2: Ribose-5-phosphate ketol-isomerase
Rpe1-2: Ribulose-5-phosphate 3-epimerase
S1,7BP: Sedoheptulose-1,7-bisphosphate
Shb17: Sedoheptulose-1,7-bisphosphatase
Tal1-2: Transaldolase
TAG: Triacylglycerols
TPP: Thiamine pyrophosphate
UPR: Unfolded protein response
XYL5P: Xylulose-5-phosphate

## References

[1] Anthony C (1982). The biochemistry of methylotrophs.

[2] Meehl MA, Stadheim TA (2014). Biopharmaceutical discovery and production in yeast. Curr Opin Biotechnol..

[3] Liu L, Yang H, Shin HD, Chen RR, Li J, Du G (2013). How to achieve high-level expression of microbial enzymes: strategies and perspectives. Bioengineered..

[4] Ma C, Agrawal G, Subramani S (2011). Peroxisome assembly: matrix and membrane protein biogenesis. J Cell Biol..

[5] Losev E, Reinke CA, Jellen J, Strongin DE, Bevis BJ, Glick BS (2006). Golgi maturation visualized in living yeast. Nature..

[6] Vogl T, Glieder A (2013). Regulation of *Pichia pastoris* promoters and its consequences for protein production. N Biotechnol..

[7] Kohlwein SD, Veenhuis M, van der Klei IJ (2013). Lipid droplets and peroxisomes: key players in cellular lipid homeostasis or a matter of fat--store ‘em up or burn’em down. Genetics..

[8] van der Klei IJ, Veenhuis M (1997). Yeast peroxisomes: function and biogenesis of a versatile cell organelle. Trends Microbiol..

[9] van der Klei IJ, Yurimoto H, Sakai Y, Veenhuis M (1763). The significance of peroxisomes in methanol metabolism in methylotrophic yeast. Biochim Biophys Acta..

[10] van Zutphen T, Baerends RJ, Susanna KA, de Jong A, Kuipers OP, Veenhuis M (2010). Adaptation of *Hansenula polymorpha* to methanol: a transcriptome analysis. BMC Genomics..

[11] Nagotu S, Krikken AM, Otzen M, Kiel JA, Veenhuis M, van der Klei IJ (2008). Peroxisome fission in *Hansenula polymorpha* requires Mdv1 and Fis1, two proteins also involved in mitochondrial fission. Traffic..

[12] Hartner FS, Glieder A (2006). Regulation of methanol utilisation pathway genes in yeasts. Microb Cell Fact..

[13] Yurimoto H, Oku M, Sakai Y (2011). Yeast methylotrophy: metabolism, gene regulation and peroxisome homeostasis. Int J Microbiol..

[14] Vanz AL, Lunsdorf H, Adnan A, Nimtz M, Gurramkonda C, Khanna N (2012). Physiological response of *Pichia pastoris* GS115 to methanol-induced high level production of the Hepatitis B surface antigen: catabolic adaptation, stress responses, and autophagic processes. Microb Cell Fact..

[15] Vanz AL, Nimtz M, Rinas U (2014). Decrease of UPR- and ERAD-related proteins in *Pichia pastoris* during methanol-induced secretory insulin precursor production in controlled fed-batch cultures. Microb Cell Fact..

[16] Liang S, Wang B, Pan L, Ye Y, He M, Han S (2012). Comprehensive structural annotation of *Pichia pastoris* transcriptome and the response to various carbon sources using deep paired-end RNA sequencing. BMC Genomics..

[17] Sauer M, Branduardi P, Gasser B, Valli M, Maurer M, Porro D (2004). Differential gene expression in recombinant *Pichia pastoris* analysed by heterologous DNA microarray hybridisation. Microb Cell Fact..

[18] Jorda J, Rojas HC, Carnicer M, Wahl A, Ferrer P, Albiol J (2014). Quantitative metabolomics and instationary ^13^C-metabolic flux analysis reveals impact of recombinant protein production on Trehalose and energy metabolism in *Pichia pastoris*. Metabolites..

[19] Rebnegger C, Graf AB, Valli M, Steiger MG, Gasser B, Maurer M (2014). In *Pichia pastoris*, growth rate regulates protein synthesis and secretion, mating and stress response. Biotechnol J..

[20] Graf A, Gasser B, Dragosits M, Sauer M, Leparc G, Tuechler T (2008). Novel insights into the unfolded protein response using *Pichia pastoris *specific DNA microarrays. BMC Genomics..

[21] Baumann K, Carnicer M, Dragosits M, Graf AB, Stadlmann J, Jouhten P (2010). A multi-level study of recombinant *Pichia pastoris* in different oxygen conditions. BMC Syst Biol..

[22] Dragosits M, Stadlmann J, Graf A, Gasser B, Maurer M, Sauer M (2010). The response to unfolded protein is involved in osmotolerance of *Pichia pastoris*. BMC Genomics..

[23] Hesketh AR, Castrillo JI, Sawyer T, Archer DB, Oliver SG (2013). Investigating the physiological response of *Pichia* (*Komagataella*) *pastoris* GS115 to the heterologous expression of misfolded proteins using chemostat cultures. Appl Microbiol Biotechnol..

[24] Dragosits M, Stadlmann J, Albiol J, Baumann K, Maurer M, Gasser B (2009). The effect of temperature on the proteome of recombinant *Pichia pastoris*. J Proteome Res..

[25] Lin XQ, Liang SL, Han SY, Zheng SP, Ye YR, Lin Y (2013). Quantitative iTRAQ LC-MS/MS proteomics reveals the cellular response to heterologous protein overexpression and the regulation of *HAC1* in *Pichia pastoris*. J Proteomics..

[26] Chung BK, Selvarasu S, Andrea C, Ryu J, Lee H, Ahn J (2010). Genome-scale metabolic reconstruction and in silico analysis of methylotrophic yeast *Pichia pastoris* for strain improvement. Microb Cell Fact..

[27] Sohn SB, Graf AB, Kim TY, Gasser B, Maurer M, Ferrer P (2010). Genome-scale metabolic model of methylotrophic yeast *Pichia pastoris* and its use for in silico analysis of heterologous protein production. Biotechnol J..

[28] Jorda J, Jouhten P, Camara E, Maaheimo H, Albiol J, Ferrer P (2012). Metabolic flux profiling of recombinant protein secreting *Pichia pastoris* growing on glucose:methanol mixtures. Microb Cell Fact..

[29] Jorda J, Suarez C, Carnicer M, ten Pierick A, Heijnen JJ, van Gulik W (2013). Glucose-methanol co-utilization in *Pichia pastoris* studied by metabolomics and in stationary ^13^C flux analysis. BMC Syst Biol..

[30] Jorda J, de Jesus SS, Peltier S, Ferrer P, Albiol J (2014). Metabolic flux analysis of recombinant *Pichia pastoris* growing on different glycerol/methanol mixtures by iterative fitting of NMR-derived ^13^C-labelling data from proteinogenic amino acids. N Biotechnol..

[31] Solà A, Jouhten P, Maaheimo H, Sánchez-Ferrando F, Szyperski T, Ferrer P (2007). Metabolic flux profiling of *Pichia pastoris* grown on glycerol/methanol mixtures in chemostat cultures at low and high dilution rates. Microbiology..

[32] Prielhofer R, Maurer M, Klein J, Wenger J, Kiziak C, Gasser B (2013). Induction without methanol: novel regulated promoters enable high-level expression in *Pichia pastoris*. Microb Cell Fact.

[33] Vogel C, Marcotte EM (2012). Insights into the regulation of protein abundance from proteomic and transcriptomic analyses. Nat Rev Genet..

[34] Lu P, Vogel C, Wang R, Yao X, Marcotte EM (2007). Absolute protein expression profiling estimates the relative contributions of transcriptional and translational regulation. Nat Biotechnol..

[35] Lee MV, Topper SE, Hubler SL, Hose J, Wenger CD, Coon JJ (2011). A dynamic model of proteome changes reveals new roles for transcript alteration in yeast. Mol Syst Biol..

[36] Kiel JA, Veenhuis M, van der Klei IJ (2006). PEX genes in fungal genomes: common, rare or redundant. Traffic..

[37] Prielhofer R, Cartwright SP, Graf AB, Valli M, Bill RM, Mattanovich D (2015). *Pichia pastoris* regulates its gene-specific response to different carbon sources at the transcriptional, rather than the translational, level. BMC Genomics..

[38] Terlecky SR, Nuttley WM, McCollum D, Sock E, Subramani S (1995). The *Pichia pastoris* peroxisomal protein PAS8p is the receptor for the C-terminal tripeptide peroxisomal targeting signal. EMBO J..

[39] Faber KN, Haima P, Gietl C, Harder W, Ab G, Veenhuis M (1994). The methylotrophic yeast *Hansenula polymorpha* contains an inducible import pathway for peroxisomal matrix proteins with an N-terminal targeting signal (PTS2 proteins). Proc Natl Acad Sci U S A..

[40] Elgersma Y, Elgersma-Hooisma M, Wenzel T, McCaffery JM, Farquhar MG, Subramani S (1998). A mobile PTS2 receptor for peroxisomal protein import in *Pichia pastoris*. J Cell Biol..

[41] Küberl A, Schneider J, Thallinger GG, Anderl I, Wibberg D, Hajek T (2011). High-quality genome sequence of *Pichia pastoris* CBS7435. J Biotechnol..

[42] Neuberger G, Maurer-Stroh S, Eisenhaber B, Hartig A, Eisenhaber F (2003). Prediction of peroxisomal targeting signal 1 containing proteins from amino acid sequence. J Mol Biol..

[43] PTS1 Predictor. http://mendel.imp.ac.at/mendeljsp/sat/pts1/PTS1predictor.jsp. Last access April 28, 2015.

[44] Wriessnegger T, Gübitz G, Leitner E, Ingolic E, Cregg J, de la Cruz B (1771). Lipid composition of peroxisomes from the yeast *Pichia pastoris* grown on different carbon sources. Biochim Biophys Acta..

[45] Luers GH, Advani R, Wenzel T, Subramani S (1998). The *Pichia pastoris* dihydroxyacetone kinase is a PTS1-containing, but cytosolic, protein that is essential for growth on methanol. Yeast..

[46] Clasquin MF, Melamud E, Singer A, Gooding JR, Xu X, Dong A (2011). Riboneogenesis in yeast. Cell..

[47] Douma AC, Veenhuis M, de Koning W, Evers M, Harder W (1985). Dihydroxyacetone synthase is localized in the peroxisomal matrix of methanol-grown *Hansenula polymorpha*. Arch Microbiol..

[48] Anderson LE, Carol AA (2004). Enzyme co-localization with rubisco in pea leaf chloroplasts. Photosynth Res..

[49] Rae BD, Long BM, Whitehead LF, Forster B, Badger MR, Price GD (2013). Cyanobacterial carboxysomes: microcompartments that facilitate CO_2_ fixation. J Mol Microbiol Biotechnol..

[50] Hahn MW (2009). Distinguishing among evolutionary models for the maintenance of gene duplicates. J Hered..

[51] Zhang J (2003). Evolution by gene duplication: an update. Trends Ecol Evol..

[52] Byun-McKay SA, Geeta R (2007). Protein subcellular relocalization: a new perspective on the origin of novel genes. Trends Ecol Evol..

[53] Daran-Lapujade P, Rossell S, van Gulik WM, Luttik MA, de Groot MJ, Slijper M (2007). The fluxes through glycolytic enzymes in *Saccharomyces cerevisiae* are predominantly regulated at posttranscriptional levels. Proc Natl Acad Sci U S A..

[54] Tibbetts AS, Sun Y, Lyon NA, Ghrist AC, Trotter PJ (2002). Yeast mitochondrial oxodicarboxylate transporters are important for growth on oleic acid. Arch Biochem Biophys..

[55] Cavero S, Vozza A, del Arco A, Palmieri L, Villa A, Blanco E (2003). Identification and metabolic role of the mitochondrial aspartate-glutamate transporter in *Saccharomyces cerevisiae*. Mol Microbiol..

[56] Kunze M, Pracharoenwattana I, Smith SM, Hartig A (1763). A central role for the peroxisomal membrane in glyoxylate cycle function. Biochim Biophys Acta..

[57] Warner JR, Mitra G, Schwindinger WF, Studeny M, Fried HM (1985). *Saccharomyces cerevisiae* coordinates accumulation of yeast ribosomal proteins by modulating mRNA splicing, translational initiation, and protein turnover. Mol Cell Biol..

[58] Puxbaum V, Mattanovich D, Gasser B (2015). Quo vadis? The challenges of recombinant protein folding and secretion in *Pichia pastoris*. Appl Microbiol Biotechnol..

[59] Hohenblum H, Gasser B, Maurer M, Borth N, Mattanovich D (2004). Effects of gene dosage, promoters, and substrates on unfolded protein stress of recombinant *Pichia pastoris*. Biotechnol Bioeng..

[60] Resina D, Bollók M, Khatri N, Valero F, Neubauer P, Ferrer P (2007). Transcriptional response of *P. pastoris* in fed-batch cultivations to *Rhizopus oryzae* lipase production reveals UPR induction. Microb Cell Fact..

[61] Ozimek P, van Dijk R, Latchev K, Gancedo C, Wang DY, van der Klei IJ (2003). Pyruvate carboxylase is an essential protein in the assembly of yeast peroxisomal oligomeric alcohol oxidase. Mol Biol Cell..

[62] Stewart MQ, Esposito RD, Gowani J, Goodman JM (2001). Alcohol oxidase and dihydroxyacetone synthase, the abundant peroxisomal proteins of methylotrophic yeasts, assemble in different cellular compartments. J Cell Sci..

[63] Waterham HR, Russell KA, Vries Y, Cregg JM (1997). Peroxisomal targeting, import, and assembly of alcohol oxidase in *Pichia pastoris*. J Cell Biol..

[64] Gudipati V, Koch K, Lienhart WD, Macheroux P (1844). The flavoproteome of the yeast *Saccharomyces cerevisiae*. Biochim Biophys Acta..

[65] Marx H, Mattanovich D, Sauer M (2008). Overexpression of the riboflavin biosynthetic pathway in *Pichia pastoris*. Microb Cell Fact..

[66] Stadlmayr G, Mecklenbrauker A, Rothmuller M, Maurer M, Sauer M, Mattanovich D (2010). Identification and characterisation of novel *Pichia pastoris* promoters for heterologous protein production. J Biotechnol..

[67] Sporty J, Lin SJ, Kato M, Ognibene T, Stewart B, Turteltaub K (2009). Quantitation of NAD^+^ biosynthesis from the salvage pathway in *Saccharomyces cerevisiae*. Yeast..

[68] Wriessnegger T, Sunga AJ, Cregg JM, Daum G (2009). Identification of phosphatidylserine decarboxylases 1 and 2 from *Pichia pastoris*. FEMS Yeast Res..

[69] Verduyn C, Postma E, Scheffers WA, van Dijken JP (1990). Physiology of *Saccharomyces cerevisiae* in anaerobic glucose-limited chemostat cultures. J Gen Microbiol..

[70] Folch J, Lees M, Sloane Stanley GH (1957). A simple method for the isolation and purification of total lipides from animal tissues. J Biol Chem..

[71] Broekhuyse RM (1968). Phospholipids in tissues of the eye. I. Isolation, characterization and quantitative analysis by two-dimensional thin-layer chromatography of diacyl and vinyl-ether phospholipids. Biochim Biophys Acta.

[72] Benjamini Y, Heller R, Yekutieli D (2009). Selective inference in complex research. Philos Trans A Math Phys Eng Sci..

[73] R project. http://www.r-project.org. Last access January 31, 2012.

[74] Pichler P, Kocher T, Holzmann J, Mazanek M, Taus T, Ammerer G (2010). Peptide labeling with isobaric tags yields higher identification rates using iTRAQ 4-plex compared to TMT 6-plex and iTRAQ 8-plex on LTQ Orbitrap. Anal Chem..

[75] Breitwieser FP, Muller A, Dayon L, Kocher T, Hainard A, Pichler P (2011). General statistical modeling of data from protein relative expression isobaric tags. J Proteome Res..

[76] Klavins K, Neubauer S, Al Chalabi A, Sonntag D, Haberhauer-Troyer C, Russmayer H (2013). Interlaboratory comparison for quantitative primary metabolite profiling in *Pichia pastoris*. Anal Bioanal Chem..

[77] Neubauer S, Haberhauer-Troyer C, Klavins K, Russmayer H, Steiger MG, Gasser B (2012). U^13^C cell extract of *Pichia pastoris*--a powerful tool for evaluation of sample preparation in metabolomics. J Sep Sci..

[78] Zamboni N, Fischer E, Sauer U (2005). FiatFlux--a software for metabolic flux analysis from ^13^C-glucose experiments. BMC Bioinformatics..

[79] Quek LE, Wittmann C, Nielsen LK, Kromer JO (2009). OpenFLUX: efficient modelling software for ^13^C-based metabolic flux analysis. Microb Cell Fact..

