# Supplementary material for: Systems-level organization of yeast methylotrophic lifestyle
Source: BMC Biol. 2015 Sep 23;13:80. doi: 10.1186/s12915-015-0186-5 (PMC4580311; doi:10.1186/s12915-015-0186-5)

Rußmayer et al., Systems-level organization of yeast methylotrophic lifestyle

Supplementary File 3

Enrichment of the peroxisomal marker protein Pex3p in the peroxisomal fraction.

Western blot analysis of subcellular fractionations from *Pichia pastoris* cultivated on complex media containing methanol as the sole carbon source is shown. Lanes were loaded with 10 µg total protein, each. (1) Homogenate; (2-5) fractions collected from a density gradient at interlayers above 17 %, 24 %, 30 % and 35 % Accudenz. Samples were analyzed with antibodies against the organelle markers Pex3p (peroxisomes) and Por1p (mitochondria). In the fraction collected from the gradient above 35 % Accudenz (lane 5) the peroxisomal marker protein Pex3p was highly enriched, whereas no enrichment of the mitochondrial marker protein Por1p was observed. Hence, this fraction was used as peroxisomal sample within this study.

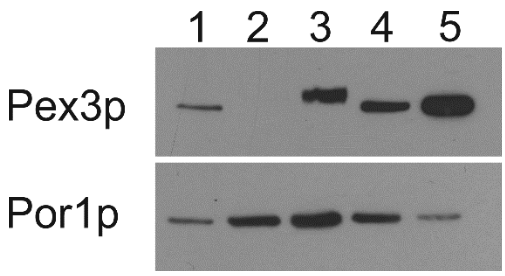

Supplement: Additional file 3: — Enrichment of the peroxisomal marker protein Pex3p in the peroxisomal fraction. (PDF 271 kb) [file 12915_2015_186_MOESM3_ESM.pdf]
